# Modular control of orchid beauty: co-expression networks orchestrate organ development and evolution in *Phalaenopsis* flower

**DOI:** 10.1007/s11103-026-01711-z

**Published:** 2026-05-28

**Authors:** Francesca Lucibelli, Angela Carfora, Marianna Varone, Gennaro Volpe, Paola Di Lillo, Sarah Maria Mazzucchiello, Giuseppe Saccone, Marco Salvemini, Serena Aceto

**Affiliations:** Department of Biology, University of Naples Federico II, Via Cintia 26, 80126 Napoli, Italy

**Keywords:** Orchidaceae, RNA-seq, Gene expression, Floral evolution, Floral development

## Abstract

The Orchidaceae family is known for its extraordinary floral diversity and unique ecological adaptations. Beyond their scientific relevance, orchids represent a major component of the ornamental plant industry due to their high commercial value. Therefore, understanding the complex molecular networks that govern orchid flower development is a fascinating and relevant challenge. Despite extensive molecular knowledge, crucial gaps remain regarding the unique developmental programs of orchids. This work aims to present a transcriptomic analysis to investigate differential gene expression across floral tissues during the early stage of flower development in *Phalaenopsis aphrodite*, in order to uncover new orchid-specific patterns. The identification of co-expression networks, combined with Gene Ontology enrichment analysis, revealed key biological pathways active across distinct floral organs. We found tissue-specific enrichment of functions related to photosynthesis, lipid metabolism, and carbohydrate metabolism. Particular emphasis was placed on genes associated with labellum traits, including pigmentation and morphology, features that are crucial for pollinator attraction and central to orchid floral evolution. The expression patterns of selected genes were validated by qPCR in both wild-type and peloric *Phalaenopsis* mutants at early developmental stages. Overall, our findings advance the understanding of the molecular regulation underlying orchid flower development and provide new insights into the evolutionary mechanisms shaping floral diversity in orchids.

**Supplementary Information:**

The online version contains supplementary material available at 10.1007/s11103-026-01711-z.

## Key message

RNA-seq analysis explores tissue-specific gene expression dynamics governing the development of specialized *Phalaenopsi*s floral traits, offering new insights into diversity and evolution of orchids.

**Supplementary Information:**

The online version contains supplementary material available at 10.1007/s11103-026-01711-z.

## Introduction

The flower is a distinctive structure unique to angiosperms and represents one of the key evolutionary innovations of this plant group, which emerged approximately 200 million years ago (Beaulieu et al. 2015; Magallon et al. 2015; Foster et al. 2017; Sauquet et al. 2017; Li et al. 2019; Benton et al. 2022). Its primary biological function is reproduction. The variation in flower pigmentation and shape contributed significantly to the rapid spread of angiosperms, the largest and most diversified group of terrestrial plants (Soltis and Soltis 2014; Zuntini et al. 2024).

Advances in sequencing technologies and the availability of genomic and transcriptomic resources have provided new insights into the genetic basis of flower evolution. In particular, several regulatory genes controlling floral development and organogenesis have been identified in model plants, such as *Arabidopsis thaliana* and *Antirrhinum majus* (O’Maoileidigh et al. 2014; Zhang et al. 2014; Klepikova et al. 2015; Yang et al. 2025).

Furthermore, comparative and evolutionary analyses of morphological and genetic traits in non-model organisms, such as orchids, have allowed the identification of conserved functions and novel key evolutionary trajectories that lead to morphological diversity (Ma et al. 2017; Song et al. 2022).

The Orchidaceae is one of the largest angiosperm families, comprising approximately 763 genera and around 28,000 species (Liu et al. 2022). Morphological and molecular phylogenetic analyses divide the Orchidaceae into five subfamilies: Apostasioideae, Vanilloideae, Cypripedioideae, Orchidoideae, and Epidendroideae (Tsai et al. 2017). The most recent common ancestor of orchids probably dates back to the Late Cretaceous in Laurasia (Perez-Escobar et al. 2024). However, the rate of orchid speciation has increased significantly over the past 5 million years, resulting in the remarkable diversity of modern orchid species. Indeed, Epidendroideae and Orchidoideae, the most recent subfamilies of orchids, are also the richest in species, likely because of the evolution of advantageous adaptive traits. These traits have enabled orchids to become a cosmopolitan group, capable of thriving in a wide variety of habitats (Perez-Escobar et al. 2024; Cozzolino and Widmer 2005).

**Fig. 1:**
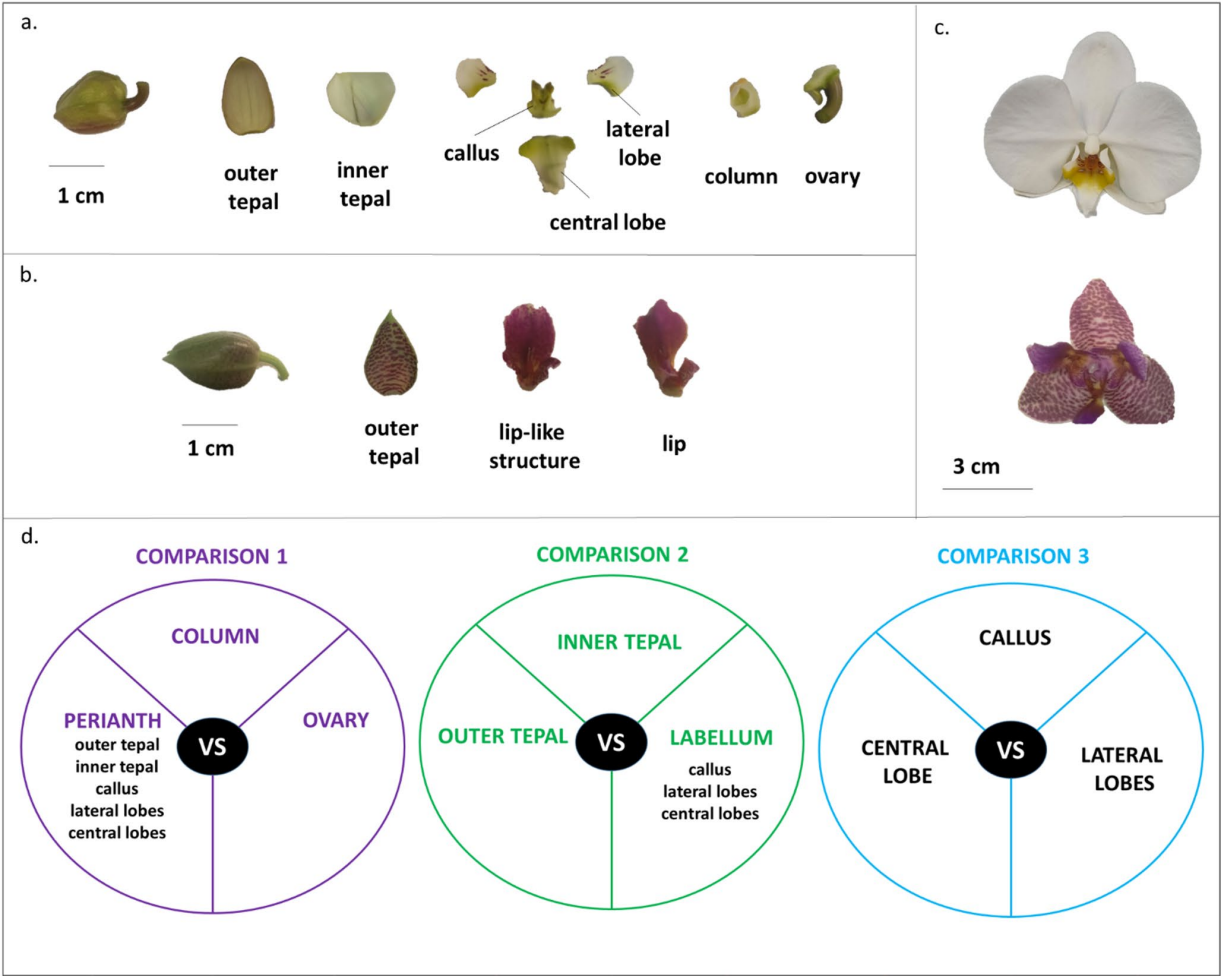
Plant material. Floral bud at stage B2 (1–1.5 cm) and its floral organs of (**a**) the wild-type *P. aphrodite* and (**b**) the peloric *Phalaenopsis* hyb. “Joy Fairy Tale”; (**c**) open flower of *P. aphrodite* (top) and *P.* hyb. “Joy Fairy Tale” (bottom); (**d**) three comparison sets used to perform *in silico* differential expression analysis: in the comparison 1, the perianth (outer and inner tepals, callus, lateral lobes, and central lobe) column and ovary were compared; in the comparison 2 the outer tepals, inner tepals, and the lip (comprising the callus, lateral lobes, and central lobe) were compared; in the comparison 3 the callus, lateral lobes, and central lobe were compared

One of the main evolutionary innovations of orchids is the establishment of the zygomorphic symmetry of the flower due to the lip differentiation and the developmental suppression of the adaxial stamens (Rudall and Bateman 2002). Zygomorphy is shared by all orchid subfamilies, except the basal Apostasioideae, which have actinomorphic flowers (Zhang et al. 2017). Despite their high morphological diversification, zygomorphic orchid flowers share a common organization (Fig. 1a-c). In the outermost whorl, there are three outer tepals; the second whorl comprises two lateral inner tepals and a median one called labellum or lip, generally with a distinctive morphology and coloration, different from the other tepals. Outer tepals, lateral inner tepals, and lip constitute the orchid perianth. All orchid flowers have female (gynoecium) and male (androecium) organs fused in the gynostemium or column. In the upper part of the gynostemium there are the pollinia, whereas in the lower part there is the ovary, whose maturation is activated after pollination (Aceto and Gaudio 2011; Rudall and Bateman 2002). In some species, the lip has evolved structural adaptations, such as calli, stelidia, and mentum, which play crucial roles in reproduction, attracting specific pollinators, securing their attachment, positioning them accurately during visits, and optimizing pollen deposition and retrieval (Pramanik et al. 2020).

The distinctive floral structure of orchids and its evolution arise from unique and sophisticated molecular pathways not found in other angiosperms, adding complexity to the classical ABCDE model that governs the specification of the different floral organs by the interaction of different classes of MADS-box transcription factors (TFs) (Theissen and Saedler 2001; De Paolo et al. 2014; Valoroso et al. 2019a).

In particular, orchid-specific regulatory mechanisms orchestrate the differentiation of the outer tepals, the lateral inner tepals, and the lip. These include the evolutionary innovations of the “orchid code”, which has given rise to models such as the Homeotic Orchid Tepal (HOT) and the Perianth code (P-code). Notably, the “orchid code” reveals how the diversity of organs within the orchid perianth is achieved through the differential expression of four class B *AP3/DEF MADS*-box genes (Mondragon-Palomino and Theissen 2008, 2009, 2011). The HOT model (Pan et al. 2011) emphasizes the importance of combinatorial interactions between MADS-box proteins during the crucial early stages of inflorescence development. Furthermore, the “P-code” (Hsu et al. 2015b; Teo et al. 2019) describes how the interaction among AP3/DEF-like, AGL6-like and PI/GLO-like proteins is essential for driving the differentiation between the tepals and the lip. In addition, other TFs are involved in the complex processes of orchid flower development. These factors (e.g., TCP, NAC, YABBY, and MYB) play key roles in determining floral organ identity, establishing symmetry, and promoting tissue differentiation (De Paolo et al. 2015; Valoroso et al. 2017, 2022; Lucibelli et al. 2020, 2021, 2025).

Although there is extensive knowledge about the molecular pathways involved in the development of orchid flowers, many crucial questions remain unanswered, particularly regarding the unique molecular patterns that distinguish orchids from other plant models. The search for homologous genes from model plants often limits studies to verifying the evolutionary conservation of pathways, which can hinder the exploration of new regulatory mechanisms unique to orchids (Zhao et al. 2023). Indeed, orchids genes often undergo functional divergence and follow distinct evolutionary trajectories, frequently related to duplication events. Among the duplicated genes, some share common expression domains, while others exhibit differential expression in association with distinct flower organs, resulting in novel functional dynamics (Zhang et al. 2017; Lucibelli et al. 2025). The complexity and plasticity of the orchid genomes are also reflected in the presence of numerous transposable elements, which can influence gene regulation and expression by moving within the genome (Cai et al. 2015; Zhang et al. 2016, 2017). Transcriptomic analyses can serve as valuable tools for uncovering and proposing new developmental programs specific to orchids by analyzing specific co-expression patterns and identifying the expression domains of orchid genes crucial to the development of specific floral organs.

In this scenario, our study aims to produce RNA-seq data publicly accessible, offering valuable insights into differential gene expression in floral tissues during the early stages of flower development in the *Phalaenopsis* orchid with particular emphasis on the labellum. Unlike previous studies that analyzed the labellum as a whole organ, we performed a fine-scale analysis by microdissecting the labellum into its distinct components (callus, lateral lobes, and central lobe). Our goal was to elucidate the specific molecular pathways driving the development of these specialized structures, which are fundamental for pollination and, consequently, for orchid evolution. Indeed, plant-insect interactions are at the base of the evolutionary success of orchids, and the specific traits of the labellum are the result of co-evolution with pollinating insects (de Jager and Peakall 2019).

We performed *in silico* differential expression analyses of floral tissues from *Phalaenopsis aphrodite* floral buds, followed by co-expression network and Gene Ontology (GO) enrichment analyses to identify gene classes showing significant differential expression across distinct floral organs. Among genes with correlated expression patterns, we identified co-expressed clusters likely representing genes involved in the same biological pathways.

From these clusters, we focused on those associated with pigmentation, a key floral trait influencing pollinator attraction. In addition, we examined TF genes known for their central roles in flower development, specifically members of the MADS, TCP, and MYB families. We selected TF genes from these families that are overexpressed in the lip and validated their expression profiles by qPCR in both wild-type *Phalaenopsis* and peloric mutants.

By making these transcriptomic data publicly available, we aim to advance the understanding of the molecular mechanisms underlying orchid flower development, providing new insights into the evolutionary processes that drive the extraordinary floral diversity of these plants.

## Materials and methods

### Plant material

The orchids utilized in this study include the wild-type *P. aphrodite* and the peloric mutant *Phalaenopsis* hybrid “Joy Fairy Tale”. All plants were cultivated under natural light and temperature in the greenhouse of the Department of Biology at the University of Naples Federico II in Naples, Italy.

Single floral buds from the wild-type *P. aphrodite* and the peloric mutant *P.* hyb. “Joy Fairy Tale” were collected before anthesis, at the B2 developmental stage (1–1.5 cm diameter size) (Fig. 1a-b). This size corresponds to the early developmental stage, as reported by previous works (Lucibelli et al. 2021; Valoroso et al. 2019b; Chen et al. 2020). For the RNA-seq experiments and *in silico* differential expression analyses, two floral buds of *P. aphrodite* were used from two different plants. Similarly, two floral buds from both *P. aphrodite* and *P*. hyb. “Joy Fairy Tale” were used for qPCR analyses.

The collected floral buds of *P. aphrodite* were dissected into outer tepals, lateral inner tepals, lip (which was further divided into callus, lateral, and central lobes), column, and ovary. The analysis of the peloric mutant *P*. hyb. “Joy Fairy Tale” was focused on perianth tissues, which include outer tepals and two lip-like organs that replace the lateral inner tepals and the lip (Fig. 1b-c). Tissue dissection was performed immediately after sampling (within 5 min) to ensure RNA integrity. The collected tissues were promptly immersed in RNAlater (Ambion, Austin, Texas) and stored at -80 °C for up to 1 week prior to RNA extraction. Total RNA was extracted from the collected tissues using the PureLink™ RNA Mini Kit (Invitrogen, Waltham, USA), following the manufacturer’s instructions.

### RNA-seq and *in silico* differential expression analysis

After extraction, the RNA from *P. aphrodite* was analyzed using the 2100 Bioanalyzer system (Agilent Technologies, Santa Clara, CA, USA) to assess fragment size, concentration, and overall quality. Poly(A)-enriched mRNA libraries were then prepared and sequenced by Novogene (Cambridge, United Kingdom) on a NovaSeq X Plus platform using a 150 bp paired-end (PE150) strategy, generating approximately 9 Gb of raw data per sample. The raw data were deposited in the NCBI database with the BioProject accession number PRJNA1364299.

Reads pre-processing, mapping, and quantification was performed following the pipeline described by Aceto et al. (2025). Although the *P. aphrodite* genome is available in public databases, it lacks functional annotation; therefore, we used the *P. equestris* genome v1.0 (ASM126359v1) as a reference, given its close phylogenetic relationship to *P. aphrodite*. Raw RNA-seq reads were first quality-filtered and trimmed to remove adapters and low-quality bases using Trimmomatic (Bolger et al. 2014). Reads were then aligned to the *P. equestris* reference genome using STAR (Dobin et al. 2013), and the resulting alignment files were processed with RSEM (Li and Dewey 2011) to obtain the count matrix.

To explore overall variance and clustering patterns among samples, Principal Component Analysis (PCA) and Biological Coefficient of Variation (BCV) analysis were performed on the log2-CPM expression matrix using the prcomp function in R.

The differential expression analysis was carried out using the edgeR package (Robinson et al. 2010). Samples were grouped according to their corresponding floral structures. *In silico *differential expression analyses were performed through three pairwise comparison sets. In the first set, the perianth (outer and inner tepals, callus, lateral lobes, and central lobe) was compared with the column and ovary. The second set compared the perianth organs outer tepals, inner tepals, and the lip (callus, lateral lobes, central lobe). The third set compared the lip substructures, specifically the callus, lateral lobes, and central lobe. The grouping scheme used for these analyses is illustrated in Fig. 1d. Genes were considered differentially expressed (DEGs) when showing an absolute log₂ fold change (|log₂FC|) > 2 and a False Discovery Rate (FDR) < 0.05.

###  Gene co-expression network analysis

Weighted Gene Co-expression Network Analysis (WGCNA) was performed in R using the WGCNA package (Langfelder and Horvath 2008) to identify co-expressed genes modules across the different floral tissues (Supplementary Table S1). GO enrichment analysis was conducted for each WGCNA module using DAVID (Huang da et al. 2009; Sherman et al. 2022) at the Biological Process category level, with the *P. equestris* gene set as background. GO terms with p-values < 0.05 were considered statistically significant.

For each module, functional annotation clustering was also performed using DAVID. This analysis groups genes with similar functional annotations by integrating multiple databases, including GO terms (Biological Process, Cellular Component, and Molecular Function), KEGG pathways, and InterPro classifications. Only functional clusters with an enrichment score > 1.3 were considered (Supplementary Table S2).

The putative promoter sequences of the genes belonging to the functional cluster of the greenyellow module associated with flavonoid biosynthesis were scanned to identify conserved transcription factor binding sites (TFBSs). These putative promoter regions, defined as 3000 bp upstream of the translation start codon of each gene, were retrieved from the *P. equestris* genome and the search for conserved *cis*-regulatory motifs was performed using PLANTPAN 4.0 (Chow et al. 2024).

### Quantitative expression analysis

The* in silico* expression profile of genes from the greenyellow module belonging to the functional cluster associated with flavonoid biosynthesis and genes from the yellow, blue, and pink modules showing high gene expression in the lip (Table 1) was validated by qPCR.

**Table 1:** List of genes selected for qPCR experiments

Gene ID	Annotation	WGCNA module
*LOC110030201*	MADS4	Yellow
*LOC110018269*	DIVARICATA-like	Yellow
*LOC110019973*	MADS6	Blue
*LOC110018630*	MADS3	Blue
*LOC110031734*	RADIALIS-like 4	Blue
*LOC110022288*	CIN8	Blue
*LOC110032610*	MADS7	Pink
*LOC110035579*	RADIALIS-like 3	Pink
*LOC110037298*	Naringenin,2-oxoglutarate3-dioxygenase-like	Greenyellow
*LOC110038151*	Flavonoid 3’-monooxygenase-like	Greenyellow
*LOC110019740*	Leucoanthocyanidin dioxygenase-like	Greenyellow
*LOC110038738*	Dihydroflavonol 4-reductase-like	Greenyellow
*LOC110024997*	Cytochrome P450	Greenyellow
*LOC110036343*	Uncharacterized subfamily of vicinal oxygenchelate	Greenyellow
*LOC110032432*	Heavy metal-associated isoprenylated plant protein 28-like	Greenyellow

For RNA-seq data validation via qPCR, total RNA was extracted from a different set of plants distinct from those used for sequencing. For each tissue dissected as described in the “plant material” section, an RNA pool was generated by combining equal quantities of total RNA from two different plants. A 500 ng of total RNA of each pool was subsequently reverse-transcribed using the LunaScript RT SuperMix Kit (NEB) according to the manufacturer’s instructions. qPCR was performed using PowerUp™ SYBR™ Green Master Mix (Applied Biosystems™) on a QuantStudio™ 5 Real-Time PCR Systems. The relative expression levels of 15 selected genes (Table 1) were evaluated in all collected tissues using *18 S* as a reference gene, as previously described (Valoroso et al. 2022; Lucibelli et al. 2025). The gene-specific primer pairs used are listed in Supplementary Table S3. The reactions were performed in technical triplicates, and the normalized relative quantity (NRQ) ± standard error of the mean (SEM) was calculated for each replicate (Hellemans et al. 2007).

ANOVA analysis followed by the Holm–Sidak post-hoc test was conducted to assess the statistical significance of the differences in NRQ among the various tissues. All statistical analyses were conducted using GraphPad Prism 9.0 (GraphPad Software, San Diego, USA).

## Results

## *In silico* gene expression profiling across the *Phalaenopsis* floral bud tissues

The raw RNA-seq reads generated from the *P. aphrodite* samples in this study have been submitted to the NCBI Sequence Read Archive (SRA), making the data freely available for the scientific community under the BioProject accession number PRJNA1364299.

*In silico* analysis of gene expression revealed distinct groups of DEGs among the outer tepals, inner tepals, callus, lip lateral lobes, lip central lobes, column, and ovary of *P. aphrodite* floral buds at the B2 developmental stage (Supplementary Figure SF1a).

Biological Coefficient of Variation (BCV) and Principal Component Analysis (PCA) (Supplementary Figures SF1c–e and SF2a–c) showed that biological replicates clustered tightly within each tissue, as also reflected in the correlation matrix (Supplementary Figure SF1b). Across all bud tissues, lip samples formed a distinct cluster apart from the outer and inner tepals, while the column and ovary were separated from the perianth organs, with the column showing the greatest distance from other groups (Supplementary Figures SF1c and SF2a). Within the perianth, the lip parts clustered separately from the outer and inner tepals, which grouped closely together (Supplementary Figures SF1d and SF2b). Further subdivision within the lip distinguished the callus from the lobes (Supplementary Figures SF1e and SF2c).

To systematically identify the molecular pathways driving the development of orchid floral structures, a hierarchical comparison strategy was used to identify gene groups differentially expressed between floral structures with distinct evolutionary origins and biological functions. Three targeted levels of biological comparisons were identified. The comparison 1 describes an organ-system differentiation, comparing the sterile perianth (tepals and labellum) against reproductive organs (column and ovary) to capture fundamental identity genes; comparison 2 describes the perianth sub-specialization, to distinguish the molecular signatures of standard tepals from the highly modified labellum; finally, comparison 3 describes the intra-labellum patterning, specifically comparing specialized structure of the callus, lateral lobes, and central lobe essential for pollinator attraction and mechanical interaction. This approach allowed a precise mapping of gene expression patterns, moving from broad organ-level identity to the fine-tuned morphological adaptations that drive orchid floral evolution.

**Fig. 2:**
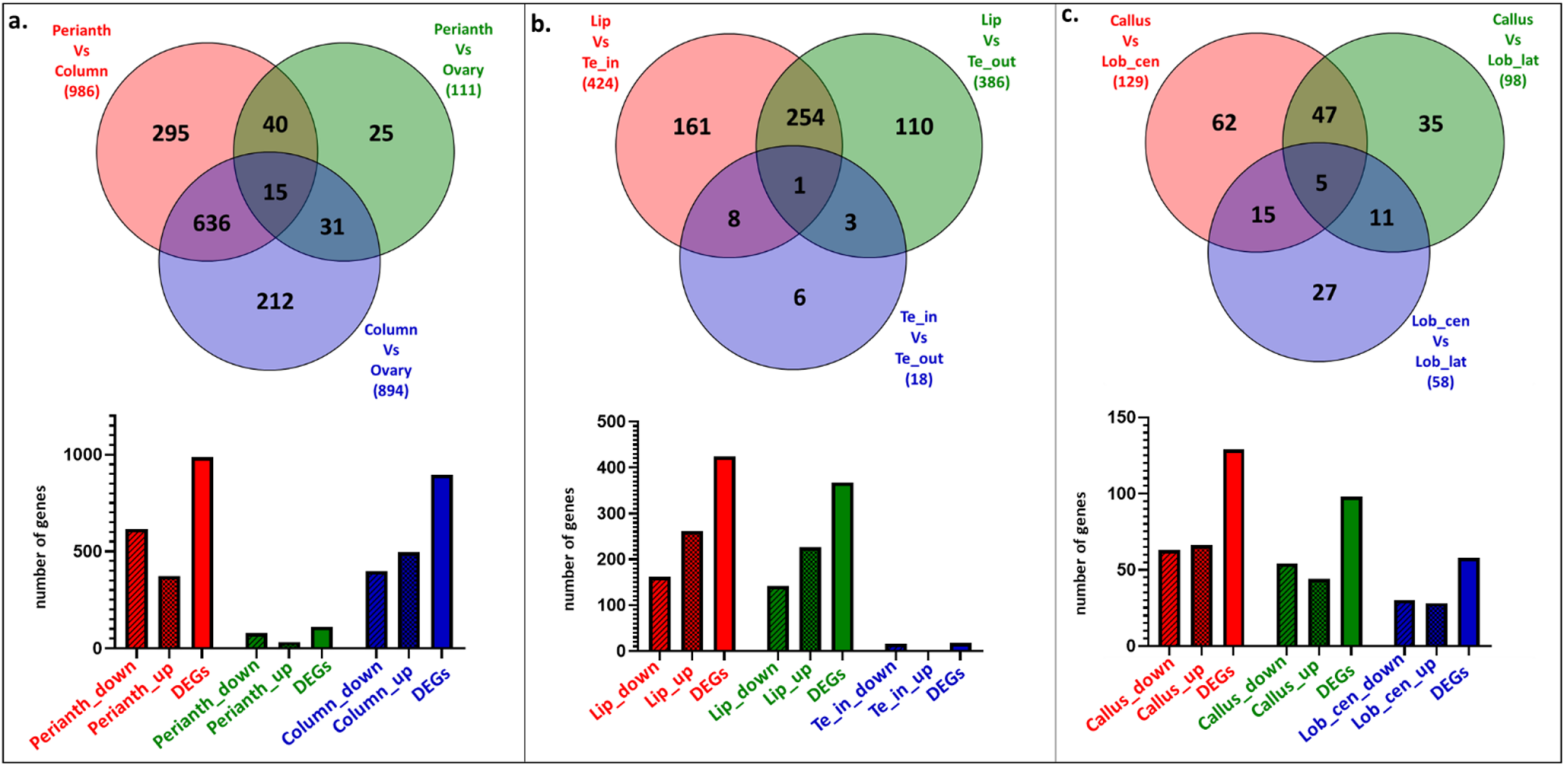
The number of DEGs identified across various tissues of the *Phalaenopsis* floral bud, based on three comparative sets. The top section features Venn diagrams displaying the number of unique and shared DEGs between the compared tissue groups: (**a**) comparison between the column, ovary, and the perianth (comprising outer tepals, inner tepals, callus, lateral lobes, and central lobe); (**b**) comparison between the outer tepals (Te_out), inner tepals (Te_in), and the lip (comprising callus, lateral lobes, and central lobe); and (**c**) comparison between the lip substructures: callus, lateral lobes (Lob_lat), and central lobe (Lob_cen). The bottom section features histograms representing the total number of genes that are up-regulated and down-regulated for each of these comparisons. DEGs displayed were selected using cutoff values (|log₂FC|) > 2 and a FDR < 0.05

Figure 2 shows the number of DEGs across the three comparisons analyzed, revealing shared and specific sets of genes that may play significant roles in common and specialized functions across tissues.

There is considerable variation between the comparisons. As expected, the highest number of DEGs (986) is found comparing the perianth with the column, followed by 894 DEGs between the column and ovary. In contrast, the number of DEGs between the perianth and ovary is smaller (111) (Fig. 2a). The number of DEGs found between the outer (Te_out) and lateral inner tepals (Te_in) is low (18), while 424 DEGs have been found between the lip and lateral inner tepals, and 368 between the lip and outer tepals (Fig. 2b). When examining the different parts of the lip, the highest number of DEGs (129) is found between the callus and central lobe (Lob_cen), whereas there are 98 DEGs between the callus and lateral lobe (Lob_lat) and 58 DEGs between the lateral lobes and central lobe (Fig. 2c).

## Detection and analysis of gene co-expression modules

**Fig. 3:**
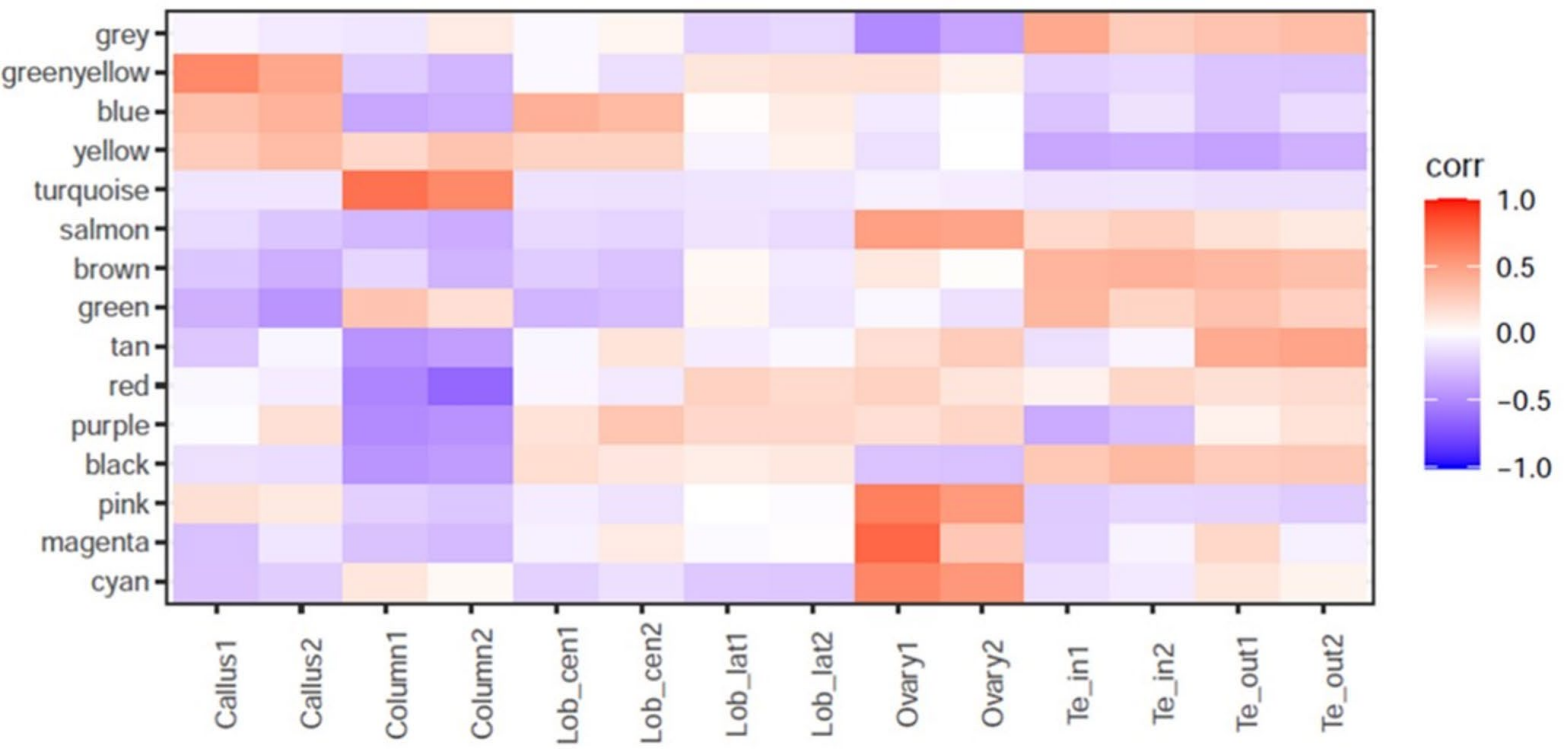
WGCNA analysis. Heatmap showing the expression profile across different tissues of genes belonging to 15 co-expression modules obtained from WGCNA analysis. The color intensity within the heatmap indicates the average level of gene expression for each module across the floral tissues analysed. Te_out (outer tepals), Te_in (inner tepals), Lob_cen (central lobe), and Lob_lat (lateral lobes), corr (correlation)

WGCNA identified 15 modules of genes that exhibit highly correlated expression in different tissues suggesting their potential involvement in the same biological processes. The method groups genes with similar expression patterns into these modules, which are then arbitrarily assigned color names to label the groups (Fig. 3). A different number of genes was found within the modules, ranging from 36 (cyan module) to 1822 (turqoise module).

While these modules often identify genes participating in the same biological pathways, they also capture broader groups of genes with diverse functions that exhibit synchronized spatial coordination; however, this does not necessarily imply that all genes within a module belong to the same biochemical pathway. This large-scale clustering provided a foundational framework for identifying functional trends and selecting candidate genes for downstream analysis.

**Fig. 4:**
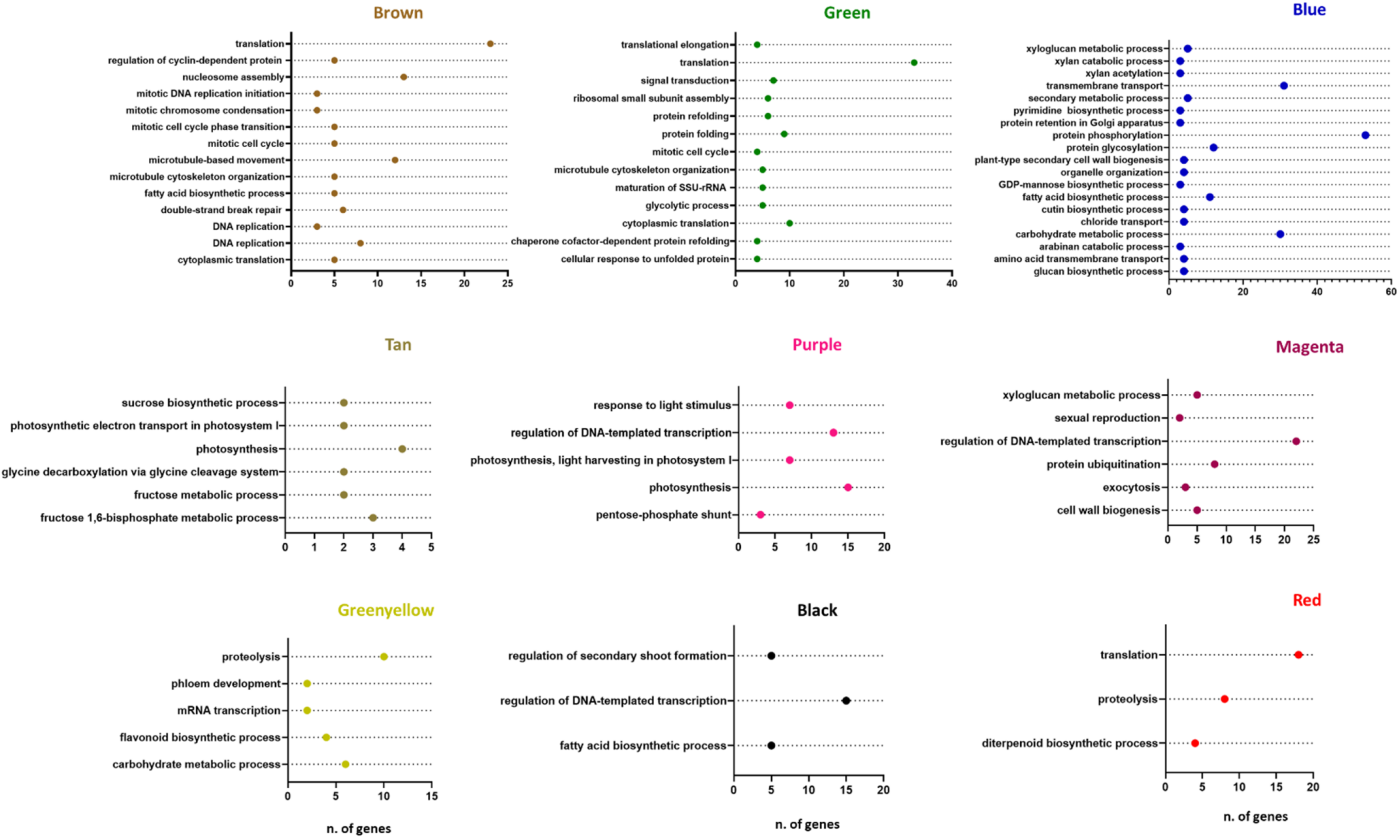
Biological process enriched in WGCNA modules. These graphs represent the results of the Gene Ontology (GO) enrichment analysis, specifically focusing on the Biological Process (BP) category level, for the genes contained within co-expression modules with high biological relevance. This analysis reveals which biological functions are statistically overrepresented in each module. On the x-axis we have the number (n) of genes and on the y-axis the GO terms

GO enrichment of the biological process was conducted on these modules to highlight the biological functions associated with these gene groups (Fig. 4 and SF3).

In addition, functional annotation clustering of the genes within each module allowed to associate genes with annotation from ontology (GO), pathways (KEGG), and protein family/domain (INTERPRO) databases that could participate in the same regulatory pathways. The genes belonging to the identified functional annotation clusters are listed in Supplementary Table S2.

The grey and cyan modules do not show enriched GO Biological Process terms, while the yellow, pink, salmon and grey modules do not show statistically significant functional clusters.

The enrichment analysis of gene co-expression modules revealed that transcription regulation is the most frequently identified biological function, characterizing the black, yellow, purple, magenta, pink, and salmon modules (Fig. 4 and SF3). Several modules, represented in Figure SF3 of the Supplementary Materials, primarily contain genes associated with basic cellular and metabolic functions and therefore do not exhibit a distinct or highly specific functional characteristic.

Specific functional enrichments were noted in several other modules. The brown module is strongly associated with cell cycle regulation, evidenced by five significant gene clusters involved in chromatin remodeling (cluster 1), translation (cluster 2), cytoskeletal reorganization (cluster 3), DNA replication and repair (cluster 4), and regulation of cell cycle phase transitions (cluster 5). Similarly, the green module primarily functions in protein translation and folding, with cluster 1 showing enrichment for ribosomal function, and clusters 2 and 3 encompassing genes related to chaperonins, heat shock proteins, and the cellular response to unfolded proteins.

The purple and tan modules share an association with photosynthesis, as indicated by the most overrepresented GO terms for biological processes. Cluster analysis within these modules further highlighted relevant biological functions, including carbon fixation, the pentose phosphate pathway, glycolysis/gluconeogenesis, starch and sucrose metabolism, chloroplast organization, and responses to light stimuli (Fig. 4, Table S2).

Other modules reflect tissue-specific functions. The magenta module, comprising genes highly expressed in the ovary, suggests a role in reproductive function. Furthermore, the greenyellow, black, and blue modules, containing genes expressed in different parts of the lip (callus and lobes), are enriched in functions related to fat biosynthesis and carbohydrate metabolism. Notably, the greenyellow module, characterized by overexpression in the callus and lateral lobes, is particularly enriched in flavonoid biosynthesis. Finally, the red module, with genes overexpressed in the tepals, is associated with diterpenoid production (Fig. 4).

## Gene cluster containing genes related to flower pigmentation

Among the gene clusters identified by DAVID, cluster 1 of the greenyellow module stands out for its significance. This cluster contains genes related to flavonoid biosynthesis, a pathway essential for anthocyanin production and, consequently, floral pigmentation. The genes of the greenyellow module show high expression in the callus and lateral lobes, floral organs that are most pigmented with purple stripes and spots (Fig. 1a) (Table S2).

This cluster comprises seven genes encoding enzymes such as oxidoreductases, dioxygenases, NADPH-dependent reductases, cytochromes, and metal-ion-binding proteins (Table 1).

**Fig. 5:**
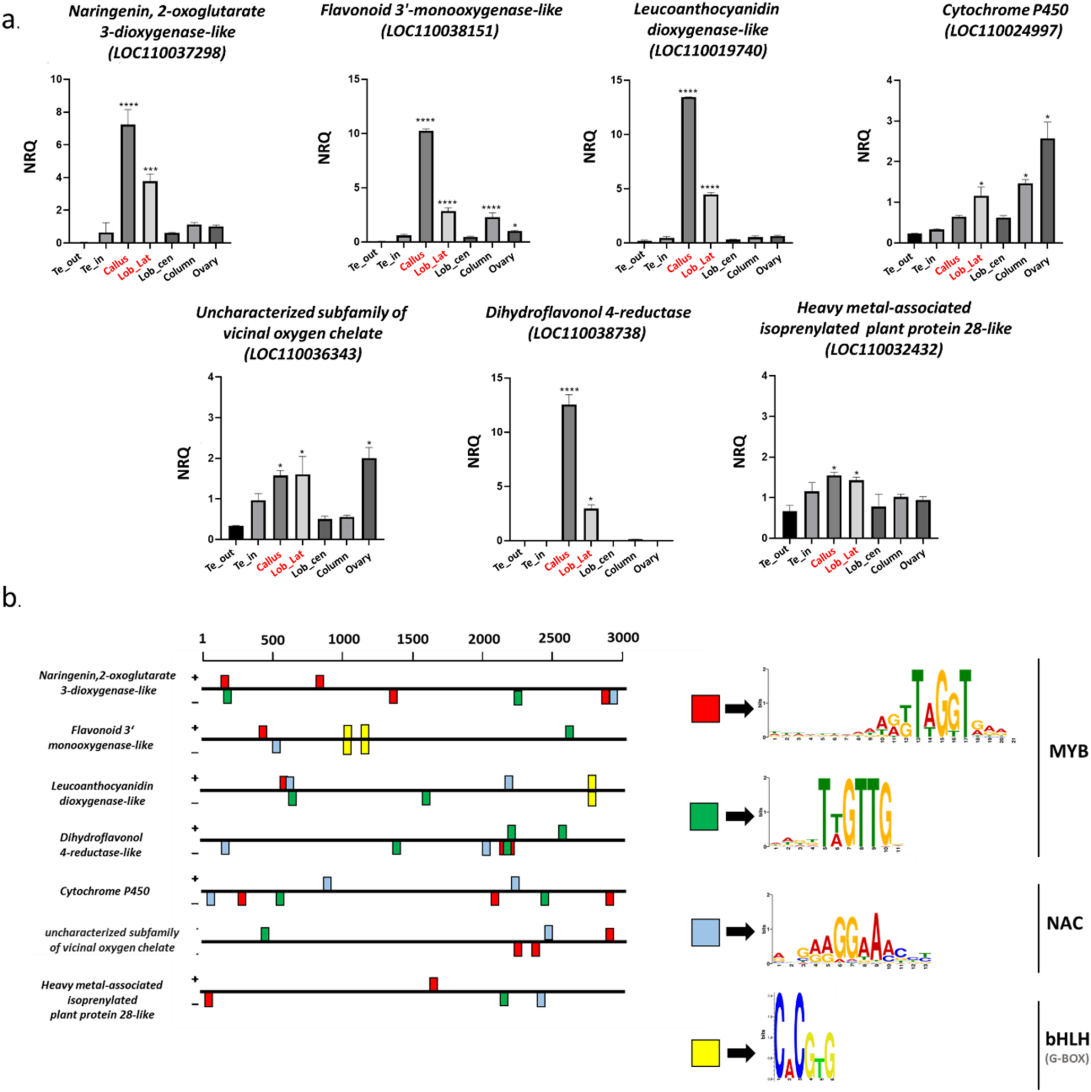
Analysis of the genes belonging to the cluster of greenyellow module related to flavonoid biosynthesis. (**a**) Relative expression of the genes belonging to the cluster 1 of greenyellow module in tissue of *P. aphrodite* at the B2 developmental stage. The expression is reported as average value of technical tripling of normalized relative quantity (NRQ). Te_out (outer tepals); Te_in (inner tepals); Lob_cen (central lobe); Lob_lat (lateral lobes). In red are indicated the lip components where genes belonging to cluster 1 show a high expression level. The vertical bars represent the SEMs of the technical replicates. The asterisks indicate the statistically significant difference of the expression compared to outer tepals. *p*-Values **** <0.0001, *p*-Values *** <0.001, *p*-Values *< 0.05. Expression levels for the displayed genes are presented on different scales; (**b**) Conserved motif within the putative promoters of genes belonging to cluster 1 of greenyellow module. The sequence logo motifs indicated by colored boxes indicated binding sites of the MYB, NAC, bHLH transcription factors

This gene cluster was subjected to quantitative PCR analysis in *P. aphrodite* buds to validate its expression profiles. The results confirmed that these genes display expression profiles consistent with their respective WGCNA module, characterized by increased expression levels in the callus and lateral lobes. Although the expression levels varied among the genes studied, the trend of elevated expression in the callus and lateral lobes was consistently observed across all analyzed genes, reinforcing the WGCNA findings (Fig. 5a).

Interestingly, in the greenyellow module, the co-occurrence of genes related to flavonoid biosynthesis with the TFs MYB, bHLH, and NAC was observed. These TF families are well-established as key regulators of the anthocyanin pathway genes (Mao et al. 2025). Specifically, the module contains two MYB114-like TFs, three bHLH-like TFs (bHLH49-like, bHLH148-like, and bHLH94-like), and one NAC domain-containing protein 86-like TF (Supplementary Table).

To provide evidence for the hypothesis that these factors could regulate the expression of genes belonging to the flavonoid biosynthesis pathway present in the studied cluster, an analysis of conserved motifs in the putative promoter regions of these genes was conducted. The 3000 bp upstream of the translation start site of the genes of interest was scanned by PlantPAN4.0 (Chow et al. 2024). The specific search for MYB, bHLH, and NAC TFBS yielded positive results in all analyzed promoters (Fig. 5b). The precise location of the TFBS is detailed in the Supplementary Table S4.

## Modules containing genes related to lip traits

An analysis was conducted focusing on the co-expression modules characterized by genes with high expression levels in the lip parts (greenyellow, blue, yellow, purple, and pink), which are critical for pollination. Within these modules, a search of genes belonging to key developmental transcription factor families was performed, specifically MADS-box and TCP, crucial for orchid development (Aceto and Gaudio 2011; De Paolo et al. 2015), and *DIVARICATA-like* and *RADIALIS-like* genes, MYB family members implicated in establishing dorsoventral symmetry and lip development (Valoroso et al. 2017, 2019b). Genes belonging to these developmental families were absent from both the purple and greenyellow modules. On the other hand, the yellow, blue, and pink modules contained genes belonging to these targeted developmental families.

In the yellow module, a MADS-box factor, *MADS4 (LOC110030201)*, belonging to the AP3/DEF clade of class B genes and implicated in lip development according to the orchid code, was identified, together with a *DIVARICATA*-like factor (*LOC110018269*).

The blue module contains two class B MADS factors: MADS3 (*LOC110018630)*, also involved in the orchid code as a regulator of lip development and belonging to the AP3/DEF clade, and MADS6 (*LOC110019973)*, which belongs to the PI/GLO clade. In addition, this module also includes the *RADIALIS-like 4* gene (*LOC110031734*) and a TCP gene, *CIN8* (*LOC110022288*).

Finally, the pink module includes a RADIALIS-like 3 factor (*LOC110035579*) and a MADS-box AGAMOUS-like factor, MADS7 (*LOC110032610*).

**Fig. 6:**
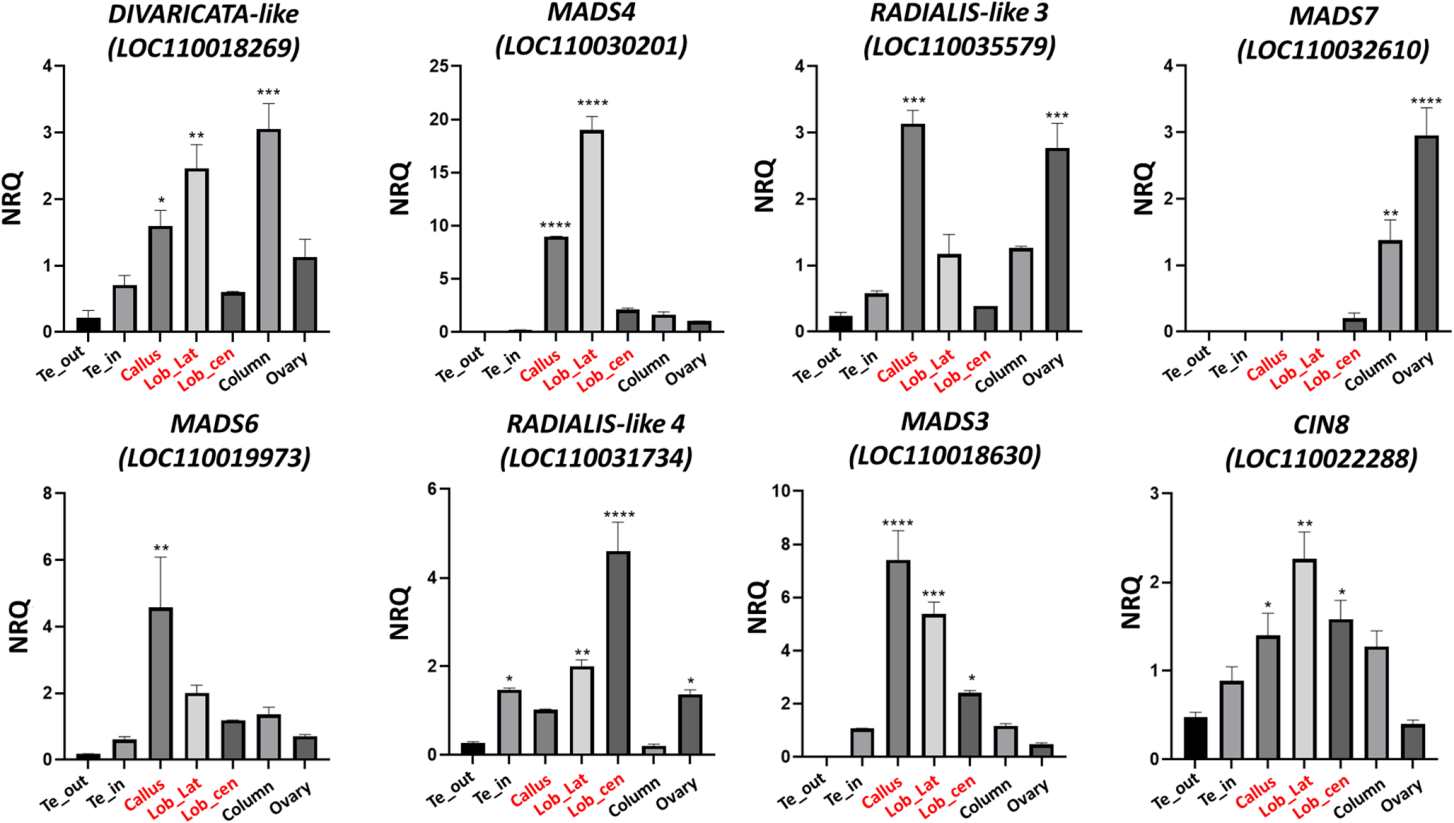
Relative expression of the genes selected in the WGCNA modules in the perianth of *P. aphrodite* at the B2 developmental stage. The expression is reported as average value of technical tripling of normalized relative quantity (NRQ). Te_out (outer tepals); Te_in (inner tepals); Lob_cen (central lobe); Lob_lat (lateral lobes). The vertical bars represent the SEMs of the technical replicates. The asterisks indicate the statistically significant difference of the expression compared to outer tepals. *p*-values *<0.05; **<0.01; ***<0.001; ****<0.0001. Expression levels for the displayed genes are presented on different scales. In red are indicated the tissue that compose the lip

qPCR analyses were conducted to verify the expression profiles of the identified TCP, MADS-box, and MYB genes belonging to the yellow, blue, and pink modules. The expression profiles largely confirmed the WGCNA results. Specifically, all analyzed genes exhibited high expression levels across the various components of the lip, including the callus, lateral lobe, and central lobe. The single exception observed was the *AGAMOUS-like* gene *MADS7*, which consistently showed expression confined exclusively to the ovary. This finding is in agreement with the general expression trend of the pink module to which this gene belongs. The *RADIALIS-like 3* gene, which also belongs to the pink module, exhibited a high level of expression in the ovary, and additionally presented high expression in the callus. The MADS-box genes *MADS4* and *MADS3* were observed to be upregulated across the different parts of the lip at the early stage of development, a finding consistent with the established orchid code model (Teo et al. 2019; Aceto and Gaudio 2011). Furthermore, the *MADS6* gene displayed high expression specifically in the callus (Hsu et al. 2015b). Regarding the TCP and MYB families, *CIN8* and *RADIALIS-like 4* exhibited an expanded expression domain across all lip parts. Finally, the *DIVARICATA-like* gene showed high expression in both the lip and the column, which is consistent with the general expression pattern of the yellow module to which it belongs (Fig. 6).

**Fig. 7:**
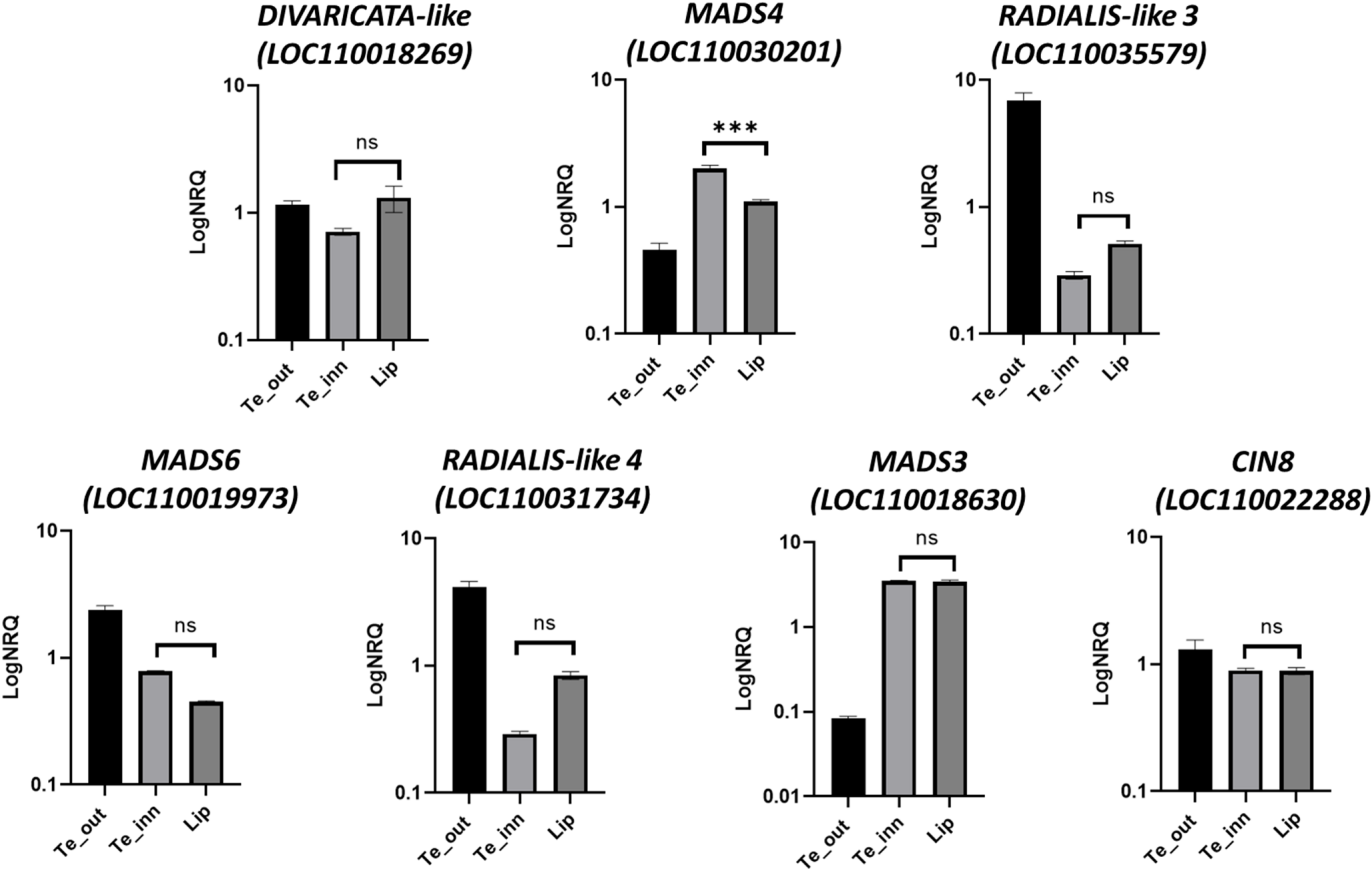
Relative expression of the genes selected in the WGCNA modules in the perianth of the peloric *Phalaenopsis* hyb. “Joy Fairy Tale” at the B2 developmental stage. The expression is reported as logarithm of normalized relative quantity (LogNRQ). The vertical bars represent the SEMs of the technical replicates. ns, not significant, *p*-Values *** <0.001. Te_out (outer tepals); Te_inn (lip-like structures that substitute the lateral inner tepals in the peloric mutant)

To support the hypothesis that the selected genes are associated with the development of the lip, the expression pattern of these genes in the peloric mutant *P.* hybrid “Joy Fairy Tale” was analysed. These mutants exhibit lip-like structures in place of the lateral inner tepals. The expression of *MADS7* was not reported in *P*. hyb “Joy Fairy Tale” perianth organs, as its expression is restricted to reproductive tissues. No significant differences were detected between the lip-like structures and the lip of the peloric flowers at the early stage of development (Fig. 7), except for *MADS4* as previously reported (Lucibelli et al. 2021). In contrast, within wild-type tissues, a statistically significant difference in expression was detected between the different parts of the lip and the internal tepals for all analyzed genes except for *MADS7* (Supplementary S5).

## Discussion

The variety of orchid flower shapes, their specialized developmental processes, unique pollination strategies, and their ability to thrive in diverse habitats make orchids a critical model for research in biodiversity conservation and evolution (Teo et al. 2019). Understanding the molecular pathways behind this variety of shapes and colors remains an open challenge. This study produces transcriptomic data aimed at identifying new key genes that may contribute to the development of the *Phalaenopsis* flower and its specialized structures. Our analysis is based on early flower tissues of *P. aphrodite*. Among all the omics tools, the transcriptomics offers insights into gene expression profiles by identifying both putative and functional genes within molecular pathways (Wang and Huo 2022).

## Differential gene expression reflects floral organ identity and orchid perianth evolution

The observed gene expression patterns across the floral organs of *P. aphrodite* align closely with the established developmental models for the Orchidaceae. BCV and PCA analyses, as well as the number of DEGs between the organ groups analysed reflect the morphological traits evolved in *Phalaenopsis* flower.

For example, the column groups very distant from the other organs showing a characteristic gene expression patterns that reflects the complexity of this organ that is typical of orchids and unique among flowering plants (Yang et al. 2021). The column exhibits species-specific variation in size and shape among orchids, and often bears additional appendages. Furthermore, the number, position, and morphology of the anthers, as well as the structural features of the pollinia, can differ significantly across species (Mondragon-Palomino and Theissen 2009).

Moreover, the finding of a comparable gene expression pattern between the outer and inner tepals, resulting in a small number of DEGs between these two tissues, is consistent with the characteristic petaloid perianth of orchids. This morphological convergence, where sepals and petals are collectively referred to as tepals, is attributed to the spatial expansion of class B MADS-box gene expression into the first floral whorl (Valoroso et al. 2019a; Chanderbali et al. 2016).

In contrast, the significantly higher number of DEGs observed between the tepals and the lip reflects that this organ is a highly specialized tepal whose unique morphology is governed by a distinct and complex molecular pathway (Hsu et al. 2021; Cheng et al. 2022). This differentiation is tightly regulated by two core genetic mechanisms centered on *DEF-like* MADS-box genes. The orchid code model proposes that lip formation is driven by high expression of clade 3 and 4 MADS-box genes coupled with reduced expression of clade 1 and 2 genes that are responsible for the formation of the outer and inner tepals (Mondragon-Palomino and Theissen 2011). In addition, the P-code model illustrates how two distinct protein complexes act antagonistically to determinate the strong differentiation between tepals and lip. The L (Lip) complex, composed of clade 3/4 class B MADS-box factors along with AGL6-like and PISTILLATA-like factors, actively promotes lip developmental identity. Conversely, the SP (Sepal/Petal) complex is formed by clade 1/2 Class B MADS-box factors together with AGL6-like and PISTILLATA-like factors, which functions to repress lip identity and favor the development of standard tepals (Teo et al. 2019).

Lastely, the observation of distinct expression patterns distinguishing the different parts of the lip highlights that each component possesses specific characteristics potentially functional for pollinator interactions (Pramanik et al. 2020). Specifically, the greater number of DEGs found between the callus and the lobes strongly suggests that the callus is a highly specialized and unique structure. The callus is characteristic of several Epidendroideae species, including *Phalaenopsis* and *Ericina pusilla*, where its functional role is to support the forelimbs of pollinating insects. Its complex identity is supported by its mixed petaloid-staminoid origin, as evidenced by its vascular supply receiving both a staminoid and several petaloid vascular bundles. This unique transcriptional and developmental signature reinforces the view of the callus as a major focus of evolutionary and functional specialization within the orchid lip (Pramanik et al. 2020; Dirks-Mulder et al. 2017).

## Functional enrichment of co-expression modules reveals pathways critical for floral organ development

The construction of a gene co-expression network allowed the identification of genes that are transcriptionally active in the same floral organs and the pathways that were enriched within the different gene modules (Burks et al. 2022). GO enrichment analysis has highlighted the biological processes that are more prevalent in different floral tissues of the bud, which may play a role in flower development.

The significant overrepresentation of transcriptional activity observed across nearly all identified modules underscores the pivotal regulatory importance of transcription factor-driven activation of specific molecular pathways during floral development and morphogenesis (Jin et al. 2017).

The enrichment of genes related to sugar metabolism in the greenyellow and blue modules, which contain genes overexpressed in the perianth and specifically the lip, is highly consistent with previous findings in orchids. RNA-sequencing studies conducted on floral organs of wild-type *Phalaenopsis* and peloric mutants have similarly shown that genes associated with starch and sucrose metabolism are among the most significantly enriched differentially expressed genes during perianth development (Huang et al. 2015).

A notable finding is the enrichment of genes related to photosynthesis within the tan and purple modules. Although photosynthesis primarily occurs in leaves, which were not analyzed in this study, in some plants, such as tobacco, other plant parts like sepals and petals can also fix CO2 during early developmental stages, albeit at lower rates than leaves (Muller et al. 2010). This observation aligns with the enrichment of photosynthetic function in the tan and purple modules and their characteristic upregulation in outer tepals, supporting the view that they maintain a leaf-like physiological identity (He et al. 2004; Muller et al. 2010).

Genes related to flavonoid production, and consequently pigmentation, are upregulated in the callus. Indeed, this biological function is particularly enriched in the greenyellow module. This observation aligns with the color patterns that start to emerge on the callus structure during the B2 floral stage. These patterns will become increasingly complex and pronounced in the open flower, which aims to attract pollinators.

Additionally, the enrichment of genes related to terpene biosynthesis in the red module suggests a functional association with the production of volatile organic compounds (VOCs) within the tepals and lip lobes. Orchids frequently emit various VOCs, many of which are terpenoids or fatty acid derivatives, as crucial chemical signals to attract specific pollinators (Hsiao et al. 2013). Indeed, genes associated with lipid metabolism were also found to be enriched in the perianth tissue, specifically within the brown, black, and blue modules (Hsiao et al. 2013). This finding suggests a potential link to the production of lipid molecules that serve as chemical cues for pollinator attraction. This hypothesis is supported by parallel evidence in other orchids. For instance, the australian orchid *Chiloglottis trapeziformis* produces a floral volatile known as chiglottone 1, which is specifically aimed at attracting male wasp pollinators. This volatile is primarily accumulated in the callus. Moreover, differential expression analyses indicate that genes associated with lipid metabolism are upregulated in the callus of this species, suggesting a connection to the tissue-specific biosynthesis of chiglottone 1 (Wong et al. 2017, 2018). In this context, the enrichment of genes associated with lipid metabolism in the blue module, which, in our *Phalaenopsis* study, is characterized by an expression trend with upregulation in the callus, is highly consistent with the parallel evidence from *C. trapeziformis.*Our data provide a framework for future metabolomic studies in *Phalaenopsis* aimed at identifying specific lipid-derived attractants in the callus, connecting gene expression profiles with ecological function.

## Validation of gene expression patterns linked to perianth organ traits

### Molecular basis of flower pigmentation: flavonoid biosynthesis, key enzymes, and conserved transcriptional regulation

The greenyellow WGCNA module, characterized by elevated expression in the callus and lateral lobes, is significantly enriched with genes governing flavonoid biosynthesis and subsequent anthocyanin production. This expression pattern directly correlates with the intensified purple spotting and streaking observed specifically in these labellum components, suggesting a molecular basis for the localized pigmentation pattern.

Anthocyanins are responsible for a broad spectrum of flower colors, ranging from pale yellow/pink to bluish violet, and their biosynthetic pathway is one of the most extensively studied. Flavonoid compounds, such as anthocyanins, contribute to the complex color patterns of angiosperms that can enhance pollination success by increasing the frequency of pollinator visit (Mao et al. 2025; Hsu et al. 2015a).

In various orchids, the expression of genes related to pigmentation, such as *chalcone synthase*, *chalcone isomerase*, *flavanone 3-hydroxylase*, and *β-carotene hydroxylase*, occurs during the bud stages when pigmentation begins to increase. A notable example is the *Rhyncholaeliocattleya* Beauty Girl KOVA, in which gene expression is significantly heightened in all parts of the perianth at the D4 bud stage (4–5 cm). This stage corresponds to the onset of pigmentation in this orchid (Li et al. 2020). This evidence is in accordance with our results, which show high expression of pigmentation-related genes in specific perianth tissues, particularly the callus, during the early developmental stage. Supporting this, our functional annotation clustering analysis successfully identified and grouped genes associated with this pathway.

The identified cluster specifically highlights the complex pathway of flavonoid biosynthesis, which is divided into the initial phenylpropanoid pathway and the subsequent dedicated flavonoid biosynthesis pathway. Flavonoids themselves comprise several major subgroups, including flavones, isoflavones, flavonols, flavanones, flavanols, anthocyanins, and proanthocyanidins, categorized based on the oxidation state and substitution of their core three-carbon heterocyclic ring. The biosynthesis of plant flavonoids involves various classes of enzymes, including oxidoreductases, transferases, hydrolases, lyases, isomerases, and ligases. In particular, oxidoreductases are the key players in this pathway, underscoring the critical role of oxidoreductase reactions in flavonoid biosynthesis (Mao et al. 2025). In the cluster identified in this work there are two oxidoreductases, *flavonoid 3’-monooxygenase-like (LOC110038151)* and *leucoanthocyanidin dioxygenase-like (LOC110019740)*, which catalyze the oxidation reactions involved in hydroxylation and desaturation in the flavonoid pathway (Wang et al. 2021). Furthermore, *naringenin*,* 2-oxoglutarate 3-dioxygenase-like (LOC110037298)* is present, which catalyzes the 3-beta-hydroxylation of 2 S-flavanones to 2R,3R-dihydroflavonols, intermediates in the biosynthesis of flavonols, anthocyanidins, catechins, and proanthocyanidins (Shirley et al. 1995). Interestingly, the cluster contains the enzyme *dihydroflavonol 4-reductase (LOC110038738)*, a central molecule in anthocyanin biosynthesis that catalyzes the NADPH-dependent reduction of dihydroflavonols (DHF) to leucoanthocyanidins, thereby regulating the accumulation of anthocyanins and proanthocyanidins (Luo et al. 2025). *Cytochrome P450 (LOC110024997)* is associated with these enzymes and catalyzes aromatic and aliphatic hydroxylations, as well as skeleton formation in flavonoid metabolism (Ayabe 2006). Finally, two genes have also been associated in this cluster, which show as their molecular function the binding to metal ions: one is a member of the subfamily of vicinal oxygen chelate metalloenzymes (VOC) (*LOC110036343)*, and the other encodes for heavy metal-associated isoprenylated plant protein 28-like (*LOC110032432)*. An association with the flavonoid pathways of these genes has not been documented, but their role in catalyzing a wide range of reactions is well established (Wang et al. 2022; de Abreu-Neto et al. 2013).

Interestingly, our results show a coexpression of the TFs MYB, bHLH, and NAC with genes involved in pigmentation, as these factors are known to be key regulators of anthocyanin pathway transcription. The sequence analysis of the putative promoter regions of the selected genes involved in the flavonoid biosynthesis revealed the presence of the conserved binding motifs for these TFs, further solidifying a possible role in gene regulation.

Previous studies report that flavonoid biosynthesis in plants is regulated by multiple TFs, among which MYB TFs play a key role. Evolutionary studies suggest a coevolutionary mechanism between MYB genes and flavonoid metabolism (Cao et al. 2020). In *A. thaliana*, MYB TFs can independently regulate the expression of specific genes in the flavonoid biosynthesis pathway or modulate flavonoid biosynthesis by forming the ternary MYB complex with bHLH factors WD40 called MBW. MYB TFs recognize AC-rich embedded motifs, while the G-box element CACGTG serves as a binding site for bHLH TFs that regulate flavonoid biosynthesis. Functional studies in *Gerbera* have demonstrated that ectopic expression of *GMYB10* leads to a significant increase in anthocyanin accumulation while mutations in *GtMYB3* in *Gentiana* result in paler flower coloration (Laitinen et al. 2008; Takashi Nakatsuka 2008).

The identification of binding motifs for these TFs in the promoters of pigmentation pathway genes uncovered in this study, along with co-expression of the corresponding regulatory genes, suggests that this regulatory model could be conserved in *Phalaenopsis*. Indeed, regarding *dihydroflavonol 4-reductase*, which was identified in our differential expression analysis, previous studies have already demonstrated its regulation by the MYB factors in *Phalaenopsis*, together with the *flavanone 3-hydroxylase PeF3H5* and anthocyanin synthase *PeANS3* (Hsu et al. 2015a). Moreover, further evidence of the conservation in orchids of this synergistic regulatory relationship between MYB factors and pigmentation-associated genes is provided by the identification of similar coordinated transcriptional regulatory networks, involving the MYB family in controlling anthocyanin accumulation in *Cymbidium goeringii* (Wang et al. 2025).

In addition to the MYB factors and the MBW complex, other TFs participate in the flavonoid biosynthetic pathway, including NAC factors. For example, in *A. thaliana*, the ANAC078 protein is associated with the expression of genes related to flavonoid biosynthesis, leading to the accumulation of anthocyanins (Morishita et al. 2009; Mao et al. 2025).

### Transcriptional control of labellum specialization: roles of MADS, TCP, and MYB factors

WGCNA modules characterized by upregulated genes in labellum include genes encoding key developmental transcription factors. The presence of these factors, which belong to the MADS, TCP, and MYB families, suggests that the complex morphological specialization of the labellum is governed by a concerted transcriptional regulatory mechanism. To test the functional correlation between gene expression and labellum morphological identity, an expression analysis was performed using a peloric hybrid that exhibits a shift toward radial symmetry, in which inner tepals are transformed into labellum-like structures. The comparable expression profiles of these critical regulators between the labellum and the labellum-like structures found in the peloric orchid mutant corroborate the hypothesis that the combinatorial expression of these specific TFs is intrinsically linked to labellum morphogenesis and specialization. Using such morphological mutants consenting to decouple organ identity from its physical position within the flower, thereby strengthening our evolutionary model of orchid floral specialization.

Overexpression of class B MADS *g*enes in the labellum was expected as described by orchid code but our results for the first time describe the expression pattern of *MADS3 (LOC110018630)*, *MADS4* (*LOC110030201)*and *MADS6* (*LOC110019973)* genes in different parts of the labellum. The pronounced expression of class B MADS genes in the early callus is particularly noteworthy, considering the evolutionary significance of this structure as a critical attachment point for pollinating insects. Furthermore, previous studies have identified additional genes with specialized expression domains in *Phalaenopsis* early callus, including the *DROOPING-LEAF 2* (*DL2*) gene, which belongs to the YABBY TF family (Lucibelli et al. 2025). This gene, which falls within the pink module in our WGCNA analysis, shows a similar expression pattern to that of the class B MADS genes, though at a reduced intensity. Importantly, it has been previously suggested that class B MADS genes and the *DL2* gene may be part of the same regulatory pathway in *Phalaenopsis* (Lucibelli et al. 2021) as this regulatory relationship has also been found in other angiosperms, such as rice (Nagasawa et al. 2003). Co-expression analysis conducted in this work provides compelling evidence to reinforce this hypothesis.

Among the MADS genes identified in the modules under analysis, it was also found the *AGAMOUS*-like factor *MADS7 (LOC110032610)* that is a D-class gene known for its role in the development of reproductive organs (Chen et al. 2012). Our *in vivo* expression results show strong expression in the ovary, as evidenced by the WGCNA analysis and the trend of the genes of the pink module to which it belongs. However, a discrepancy was noted: while the pink module genes collectively display an expression signal in the callus, this specific expression was not detected for *MADS7* in the subsequent *in vivo *validation. This lack of detection is likely due to the expression level being too low in the callus to be detectable by the quantitative expression analysis, despite being captured by *in silico* differential expression analysis. The detection of class D MADS genes, typically associated with reproductive organ development, within the callus is not unexpected, as this expression pattern reflects the ontogenetic origin of the callus from sterile, primitive stamen-like complexes (Pramanik et al. 2020; Dirks-Mulder et al. 2017).

Belonging to the *TCP* gene family, *CIN 8 (LOC110022288)* displays an increase in expression within the labellum relative to the other floral organs. This specific expression pattern points towards a key function in driving cell cycle regulation and proliferation, which is indispensable for orchestrating the complex and rapid growth required to establish the distinct shape and specialized features of the labellum (Lin et al. 2016).

The expressed profile of selected *DIVARICATA*-like and *RADIALIS*-like genes reflect reduntant roles of these genes as described by prior research on *DIV*- and *RAD*-like gene expression in *Orchis italica* (Valoroso et al. 2017). Specifically, *DIV*-like gene (*LOC110018269*) shows a significant expression in the column in addition to the labellum validating *in silico* analysis. Notably, *DIVARICATA*-like genes exhibit a pleiotropic role in many angiosperms, also because this family of genes is characterized by numerous duplication events that could induce the acquisition of new functions (Howarth and Donoghue 2009; Valoroso et al. 2017). On the other hand, the identification of *RADIALIS-like* genes in lip-associated modules is consistent with their crucial role in establishing the dorsal identity of the flower and the bilateral symmetry. Although the lip is developmentally the uppermost perianth organ, its physical position is inferior due to resupination, a 180° rotation that occurs during the development of many orchid flowers. Consequently, these genes are hypothesized to specifically regulate the development of this specialized, dorsalized organ.

## Conclusions

This study enhances our understanding of the molecular pathways that regulate *Phalaenopsis* development by providing a comprehensive, publicly accessible transcriptomic dataset that encompasses all floral tissues at an early developmental stage, including fine-scale resolution of the unique lip morphology through dissection of its specialized parts. The resulting gene co-expression network analysis highlighted several predominant biological functions enriched within different floral organs. We found that the labellum exhibits strong enrichment for flavonoid biosynthesis, directly correlating with pigmentation patterns crucial for pollinator attraction and evolution. Other enriched functions across the tissues include photosynthesis and the metabolism of sugars and lipids. Crucially, the consistent enhancement of transcriptional functions across nearly all modules underscores the pivotal role of transcription factor-driven regulation in activating developmental pathways. The identification of key members of prominent TF families (MADS, TCP, and MYB) among differentially expressed genes confirms their central significance in floral development and diversification. By integrating co-expression network analysis with developmental transcriptomics, this study offers novel insights into the regulatory genetic mechanisms underlying floral diversification in orchids. Collectively, these results lay the groundwork for future functional studies aimed at elucidating the genetic basis of orchid floral traits and their evolutionary adaptations, paving the way for future advancements in orchid research and conservation.

## Supplementary Information

Below is the link to the electronic supplementary material.

Supplementary List

Supplementary Material S2

Supplementary Material S5

Supplementary Material S3

Supplementary Material S1

Supplementary Material SF2

Supplementary Material SF3

Supplementary Material SF1

Supplementary Material S4

## Data Availability

The data presented in this study are available in the manuscript and its Supplementary Material. RNA-seq data from *Phalaenopsis aphrodite* bud (1-1,5 cm) tissue: outer tepals, lateral inner tepals, lip (which was further divided into callus, lateral, and central lobes), column, and ovary are available in the NCBI database with the BioProject accession number PRJNA1364299 (https://www.ncbi.nlm.nih.gov/bioproject/PRJNA1364299/).

## References

[CR1] Aceto S, Gaudio L (2011) The MADS and the beauty: genes involved in the development of orchid flowers. Curr Genomics 12(5):342–356. 10.2174/138920211796429754 22294877 10.2174/138920211796429754 PMC3145264

[CR2] Aceto S, Perrini S, Varone M, Lucibelli F, Volpe G, Di Lillo P, Carfora A, Mazzucchiello SM, Saccone G, Salvemini M (2025) Identification of sex-specific and sex-biased transcripts for genetic sexing. Methods Mol Biol 2935:273–298. 10.1007/978-1-0716-4583-3_12 40828283 10.1007/978-1-0716-4583-3_12

[CR3] Ayabe S, Akashi T (2006) Cytochrome P450s in flavonoid metabolism. Phytochem Rev 5:271–282

[CR4] Beaulieu JM, O’Meara BC, Crane P, Donoghue MJ (2015) Heterogeneous rates of molecular evolution and diversification could explain the triassic age estimate for angiosperms. Syst Biol 64(5):869–878. 10.1093/sysbio/syv027 25944476 10.1093/sysbio/syv027

[CR5] Benton MJ, Wilf P, Sauquet H (2022) The angiosperm terrestrial revolution and the origins of modern biodiversity. New Phytol 233(5):2017–2035. 10.1111/nph.17822 34699613 10.1111/nph.17822

[CR6] Bolger AM, Lohse M, Usadel B (2014) Trimmomatic: a flexible trimmer for Illumina sequence data. Bioinformatics 30(15):2114–2120. 10.1093/bioinformatics/btu170 24695404 10.1093/bioinformatics/btu170 PMC4103590

[CR7] Burks DJ, Sengupta S, De R, Mittler R, Azad RK (2022) The Arabidopsis gene co-expression network. Plant direct 6(4):e396. 10.1002/pld3.396 35492683 10.1002/pld3.396 PMC9039629

[CR8] Cai J, Liu X, Vanneste K, Proost S, Tsai WC, Liu KW, Chen LJ, He Y, Xu Q, Bian C, Zheng Z, Sun F, Liu W, Hsiao YY, Pan ZJ, Hsu CC, Yang YP, Hsu YC, Chuang YC, Dievart A, Dufayard JF, Xu X, Wang JY, Wang J, Xiao XJ, Zhao XM, Du R, Zhang GQ, Wang M, Su YY, Xie GC, Liu GH, Li LQ, Huang LQ, Luo YB, Chen HH, Van de Peer Y, Liu ZJ (2015) The genome sequence of the orchid equestris. Nat Genet 47(1):65–72. 10.1038/ng.3149 25420146 10.1038/ng.3149

[CR9] Cao Y, Li K, Li Y, Zhao X, Wang L (2020) MYB transcription factors as regulators of secondary metabolism in plants. Biology 9(3):61 32213912 10.3390/biology9030061 PMC7150910

[CR10] Chanderbali AS, Berger BA, Howarth DG, Soltis PS, Soltis DE (2016) Evolving ideas on the origin and evolution of flowers: new perspectives in the genomic era. Genetics 202(4):1255–1265. 10.1534/genetics.115.182964 27053123 10.1534/genetics.115.182964 PMC4905540

[CR12] Chen YY, Lee PF, Hsiao YY, Wu WL, Pan ZJ, Lee YI, Liu KW, Chen LJ, Liu ZJ, Tsai WC (2012) C- and D-class MADS-box genes from Phalaenopsis equestris (Orchidaceae) display functions in gynostemium and ovule development. Plant Cell Physiol 53(6):1053–1067. 10.1093/pcp/pcs048 22499266 10.1093/pcp/pcs048

[CR11] Chen YY, Hsiao YY, Chang SB, Zhang D, Lan SR, Liu ZJ, Tsai WC (2020) Genome-wide identification of YABBY genes in orchidaceae and their expression patterns in phalaenopsis orchid. Genes. 10.3390/genes11090955 10.3390/genes11090955 PMC7563141 32825004

[CR13] Cheng H, Xie X, Ren M, Yang S, Zhao X, Mahna N, Liu Y, Xu Y, Xiang Y, Chai H, Zheng L, Ge H, Jia R (2022) Characterization of three SEPALLATA-Like MADS-box genes associated with floral development in Paphiopedilum henryanum (Orchidaceae). Front Plant Sci 13:916081. 10.3389/fpls.2022.916081 35693163 10.3389/fpls.2022.916081 PMC9178235

[CR14] Chow CN, Yang CW, Wu NY, Wang HT, Tseng KC, Chiu YH, Lee TY, Chang WC (2024) PlantPAN 4.0: updated database for identifying conserved non-coding sequences and exploring dynamic transcriptional regulation in plant promoters. Nucleic Acids Res 52(D1):D1569–D1578. 10.1093/nar/gkad945 37897338 10.1093/nar/gkad945 PMC10767843

[CR15] Cozzolino S, Widmer A (2005) Orchid diversity: an evolutionary consequence of deception? Trends Ecol Evol 20(9):487–494. 10.1016/j.tree.2005.06.004 10.1016/j.tree.2005.06.004 16701425

[CR16] de Abreu-Neto JB, Turchetto-Zolet AC, de Oliveira LF, Zanettini MH, Margis-Pinheiro M (2013) Heavy metal-associated isoprenylated plant protein (HIPP): characterization of a family of proteins exclusive to plants. FEBS J 280(7):1604–1616. 10.1111/febs.12159 23368984 10.1111/febs.12159

[CR17] de Jager ML, Peakall R (2019) Experimental examination of pollinator-mediated selection in a sexually deceptive orchid. Ann Botany 123(2):347–354. 10.1093/aob/mcy083 29878057 10.1093/aob/mcy083 PMC6344214

[CR19] De Paolo S, Salvemini M, Gaudio L, Aceto S (2014) De novo transcriptome assembly from inflorescence of Orchis italica: analysis of coding and non-coding transcripts. PLoS ONE 9(7):e102155. 10.1371/journal.pone.0102155 25025767 10.1371/journal.pone.0102155 PMC4099010

[CR18] De Paolo S, Gaudio L, Aceto S (2015) Analysis of the TCP genes expressed in the inflorescence of the orchid Orchis italica. Sci Rep 5:16265. 10.1038/srep16265 26531864 10.1038/srep16265 PMC4632031

[CR20] Dirks-Mulder A, Butot R, van Schaik P, Wijnands JW, van den Berg R, Krol L, Doebar S, van Kooperen K, de Boer H, Kramer EM, Smets EF, Vos RA, Vrijdaghs A, Gravendeel B (2017) Exploring the evolutionary origin of floral organs of Erycina pusilla, an emerging orchid model system. BMC Evol Biol 17(1):89. 10.1186/s12862-017-0938-7 28335712 10.1186/s12862-017-0938-7 PMC5364718

[CR21] Dobin A, Davis CA, Schlesinger F, Drenkow J, Zaleski C, Jha S, Batut P, Chaisson M, Gingeras TR (2013) STAR: ultrafast universal RNA-seq aligner. Bioinformatics 29(1):15–21. 10.1093/bioinformatics/bts635 23104886 10.1093/bioinformatics/bts635 PMC3530905

[CR22] Foster CSP, Sauquet H, van der Merwe M, McPherson H, Rossetto M, Ho SYW (2017) Evaluating the impact of genomic data and priors on bayesian estimates of the angiosperm evolutionary timescale. Syst Biol 66(3):338–351. 10.1093/sysbio/syw086 27650175 10.1093/sysbio/syw086

[CR23] He C, Munster T, Saedler H (2004) On the origin of floral morphological novelties. FEBS Lett 567(1):147–151. 10.1016/j.febslet.2004.02.090 15165908 10.1016/j.febslet.2004.02.090

[CR24] Hellemans J, Mortier G, De Paepe A, Speleman F, Vandesompele J (2007) qBase relative quantification framework and software for management and automated analysis of real-time quantitative PCR data. Genome Biol 8(2):R19. 10.1186/gb-2007-8-2-r19 17291332 10.1186/gb-2007-8-2-r19 PMC1852402

[CR25] Howarth DG, Donoghue MJ (2009) Duplications and expression of DIVARICATA-like genes in dipsacales. Mol Biol Evol 26(6):1245–1258. 10.1093/molbev/msp051 19289599 10.1093/molbev/msp051

[CR26] Hsiao YY, Huang TH, Fu CH, Huang SC, Chen YJ, Huang YM, Chen WH, Tsai WC, Chen HH (2013) Transcriptomic analysis of floral organs from Phalaenopsis orchid by using oligonucleotide microarray. Gene 518(1):91–100. 10.1016/j.gene.2012.11.069 23262337 10.1016/j.gene.2012.11.069

[CR27] Hsu CC, Chen YY, Tsai WC, Chen WH, Chen HH (2015a) Three R2R3-MYB transcription factors regulate distinct floral pigmentation patterning in Phalaenopsis spp. Plant Physiol 168(1):175–191. 10.1104/pp.114.254599 25739699 10.1104/pp.114.254599 PMC4424010

[CR28] Hsu H, Hsu W, Lee Y, Mao W, Yang J, Li J, Yang C (2015b) Model for perianth formation in orchids. Nat Plants 1

[CR29] Hsu HF, Chen WH, Shen YH, Hsu WH, Mao WT, Yang CH (2021) Multifunctional evolution of B and AGL6 MADS box genes in orchids. Nat Commun 12(1):902. 10.1038/s41467-021-21229-w 33568671 10.1038/s41467-021-21229-w PMC7876132

[CR31] Huang JZ, Lin CP, Cheng TC, Chang BC, Cheng SY, Chen YW, Lee CY, Chin SW, Chen FC (2015) A de novo floral transcriptome reveals clues into Phalaenopsis orchid flower development. PLoS ONE 10(5):e0123474. 10.1371/journal.pone.0123474 25970572 10.1371/journal.pone.0123474 PMC4430480

[CR30] Huang da W, Sherman BT, Lempicki RA (2009) Systematic and integrative analysis of large gene lists using DAVID bioinformatics resources. Nat Protoc 4(1):44–57. 10.1038/nprot.2008.211 19131956 10.1038/nprot.2008.211

[CR32] Jin J, Tian F, Yang DC, Meng YQ, Kong L, Luo J, Gao G (2017) PlantTFDB 4.0: toward a central hub for transcription factors and regulatory interactions in plants. Nucleic Acids Res 45(D1):D1040–D1045. 10.1093/nar/gkw982 27924042 10.1093/nar/gkw982 PMC5210657

[CR33] Klepikova AV, Logacheva MD, Dmitriev SE, Penin AA (2015) RNA-seq analysis of an apical meristem time series reveals a critical point in Arabidopsis thaliana flower initiation. BMC Genomics 16(1):466. 10.1186/s12864-015-1688-9 26084880 10.1186/s12864-015-1688-9 PMC4470339

[CR34] Laitinen RA, Ainasoja M, Broholm SK, Teeri TH, Elomaa P (2008) Identification of target genes for a MYB-type anthocyanin regulator in Gerbera hybrida. J Exp Bot 59(13):3691–3703. 10.1093/jxb/ern216 18725377 10.1093/jxb/ern216 PMC2561154

[CR35] Langfelder P, Horvath S (2008) WGCNA: an R package for weighted correlation network analysis. BMC Bioinform 9:559. 10.1186/1471-2105-9-559 19114008 10.1186/1471-2105-9-559 PMC2631488

[CR36] Li B, Dewey CN (2011) RSEM: accurate transcript quantification from RNA-Seq data with or without a reference genome. BMC Bioinform 12:323. 10.1186/1471-2105-12-323 21816040 10.1186/1471-2105-12-323 PMC3163565

[CR38] Li HT, Yi TS, Gao LM, Ma PF, Zhang T, Yang JB, Gitzendanner MA, Fritsch PW, Cai J, Luo Y, Wang H, van der Bank M, Zhang SD, Wang QF, Wang J, Zhang ZR, Fu CN, Yang J, Hollingsworth PM, Chase MW, Soltis DE, Soltis PS, Li DZ (2019) Origin of angiosperms and the puzzle of the Jurassic gap. Nat plants 5(5):461–470. 10.1038/s41477-019-0421-0 31061536 10.1038/s41477-019-0421-0

[CR37] Li BJ, Zheng B, Wang JY, Tsai WC, Lu HC, Zou LH, Wan X, Zhang DY, Qiao HJ, Liu ZJ, Wang Y (2020) New insight into the molecular mechanism of colour differentiation among floral segments in orchids. Commun Biol 3 (89) 10.1038/s42003-020-0821-8 PMC7048853 32111943

[CR39] Lin YF, Chen YY, Hsiao YY, Shen CY, Hsu JL, Yeh CM, Mitsuda N, Ohme-Takagi M, Liu ZJ, Tsai WC (2016) Genome-wide identification and characterization of TCP genes involved in ovule development of Phalaenopsis equestris. J Exp Bot 67(17):5051–5066. 10.1093/jxb/erw273 27543606 10.1093/jxb/erw273 PMC5014156

[CR40] Liu C, Leng J, Li Y, Ge T, Li J, Chen Y, Guo C, Qi J (2022) A spatiotemporal atlas of organogenesis in the development of orchid flowers. Nucleic Acids Res. 10.1093/nar/gkac773 36095130 10.1093/nar/gkac773 PMC9508851

[CR42] Lucibelli F, Valoroso MC, Aceto S (2020) Radial or bilateral? The molecular basis of floral symmetry. Genes. 10.3390/genes11040395 10.3390/genes11040395 PMC7230197 32268578

[CR43] Lucibelli F, Valoroso MC, Theissen G, Nolden S, Mondragon-Palomino M, Aceto S (2021) Extending the toolkit for beauty: differential co-expression of DROOPING LEAF-Like and class B MADS-Box genes during Phalaenopsis flower development. Int J Mol Sci. 10.3390/ijms22137025 10.3390/ijms22137025 PMC8268020 34209912

[CR41] Lucibelli F, Carfora A, Becker A, Ehlers K, Aceto S (2025) Evolutionary dynamics of Orchid DL paralogs: gene duplication, functional divergence, and expression patterns across Orchid subfamilies. BMC Plant Biol 25(1):889. 10.1186/s12870-025-06936-6 40629286 10.1186/s12870-025-06936-6 PMC12235851

[CR44] Luo S, Wang S, Yang L, Luo K, Cheng J, Ning Y, Dong Y, Wang W (2025) A comprehensive evolutionary analysis of the dihydroflavonol 4-Reductase (DFR) gene family in plants: insights from 237 species. Genes. 10.3390/genes16040396 10.3390/genes16040396 PMC12027299 40282359

[CR45] Ma Q, Zhang W, Xiang QY (2017) Evolution and developmental genetics of floral display—a review of progress. J Syst Evol 55(6):487–515

[CR46] Magallon S, Gomez-Acevedo S, Sanchez-Reyes LL, Hernandez-Hernandez T (2015) A metacalibrated time-tree documents the early rise of flowering plant phylogenetic diversity. New Phytol 207(2):437–453. 10.1111/nph.13264 25615647 10.1111/nph.13264

[CR47] Mao Y, Luo J, Cai Z (2025) Biosynthesis and regulatory mechanisms of plant flavonoids: a review. Plants. 10.3390/plants14121847 10.3390/plants14121847 PMC12196789 40573835

[CR48] Mondragon-Palomino M, Theissen G (2008) MADS about the evolution of orchid flowers. Trends Plant Sci 13(2):51–59. 10.1016/j.tplants.2007.11.007 18262819 10.1016/j.tplants.2007.11.007

[CR49] Mondragon-Palomino M, Theissen G (2009) Why are orchid flowers so diverse? Reduction of evolutionary constraints by paralogues of class B floral homeotic genes. Ann Botany 104(3):583–594. 10.1093/aob/mcn258 19141602 10.1093/aob/mcn258 PMC2720651

[CR50] Mondragon-Palomino M, Theissen G (2011) Conserved differential expression of paralogous DEFICIENS- and GLOBOSA-like MADS-box genes in the flowers of Orchidaceae: refining the ‘orchid code’. Plant J Cell Mol Biol 66(6):1008–1019. 10.1111/j.1365-313X.2011.04560.x 10.1111/j.1365-313X.2011.04560.x 21435045

[CR51] Morishita T, Kojima Y, Maruta T, Nishizawa-Yokoi A, Yabuta Y, Shigeoka S (2009) Arabidopsis NAC transcription factor, ANAC078, regulates flavonoid biosynthesis under high-light. Plant Cell Physiol 50(12):2210–2222. 10.1093/pcp/pcp159 19887540 10.1093/pcp/pcp159

[CR52] Muller GL, Drincovich MF, Andreo CS, Lara MV (2010) Role of photosynthesis and analysis of key enzymes involved in primary metabolism throughout the lifespan of the tobacco flower. J Exp Bot 61(13):3675–3688. 10.1093/jxb/erq187 20591899 10.1093/jxb/erq187

[CR53] Nagasawa N, Miyoshi M, Sano Y, Satoh H, Hirano H, Sakai H, Nagato Y (2003) SUPERWOMAN1 and DROOPING LEAF genes control floral organ identity in rice. Development 130(4):705–718. 10.1242/dev.00294 12506001 10.1242/dev.00294

[CR54] O’Maoileidigh DS, Graciet E, Wellmer F (2014) Gene networks controlling Arabidopsis thaliana flower development. New Phytol 201(1):16–30. 10.1111/nph.12444 23952532 10.1111/nph.12444

[CR55] Pan Z, Cheng C, Tsai W, Chung M, Chen W, Hu J, HH. C (2011) The duplicated B-class MADS-Box genes display dualistic characters in orchid floral organ identity and growth. Plant Cell Biol 52(9):1515–1531 10.1093/pcp/pcr092 21757456

[CR56] Perez-Escobar OA, Bogarin D, Przelomska NAS, Ackerman JD, Balbuena JA, Bellot S, Buhlmann RP, Cabrera B, Cano JA, Charitonidou M, Chomicki G, Clements MA, Cribb P, Fernandez M, Flanagan NS, Gravendeel B, Hagsater E, Halley JM, Hu AQ, Jaramillo C, Mauad AV, Maurin O, Muntz R, Leitch IJ, Li L, Negrao R, Oses L, Phillips C, Rincon M, Salazar GA, Simpson L, Smidt E, Solano-Gomez R, Parra-Sanchez E, Tremblay RL, van den Berg C, Tamayo BSV, Zuluaga A, Zuntini AR, Chase MW, Fay MF, Condamine FL, Forest F, Nargar K, Renner SS, Baker WJ, Antonelli A (2024) The origin and speciation of orchids. New Phytol 242(2):700–716. 10.1111/nph.19580 38382573 10.1111/nph.19580

[CR57] Pramanik D, Dorst N, Meesters N, Spaans M, Smets E, Welten M, Gravendeel B (2020) Evolution and development of three highly specialized floral structures of bee-pollinated Phalaenopsis species. Evodevo 11:16. 10.1186/s13227-020-00160-z 32793330 10.1186/s13227-020-00160-z PMC7418404

[CR58] Robinson MD, McCarthy DJ, Smyth GK (2010) edgeR: a Bioconductor package for differential expression analysis of digital gene expression data. Bioinformatics 26(1):139–140. 10.1093/bioinformatics/btp616 19910308 10.1093/bioinformatics/btp616 PMC2796818

[CR59] Rudall PJ, Bateman RM (2002) Roles of synorganisation, zygomorphy and heterotopy in floral evolution: the gynostemium and labellum of orchids and other lilioid monocots. Biol Rev Camb Philos Soc 77(3):403–441. 10.1017/s1464793102005936 12227521 10.1017/s1464793102005936

[CR60] Sauquet H, von Balthazar M, Magallon S, Doyle JA, Endress PK, Bailes EJ, Barroso de Morais E, Bull-Herenu K, Carrive L, Chartier M, Chomicki G, Coiro M, Cornette R, El Ottra JHL, Epicoco C, Foster CSP, Jabbour F, Haevermans A, Haevermans T, Hernandez R, Little SA, Lofstrand S, Luna JA, Massoni J, Nadot S, Pamperl S, Prieu C, Reyes E, Dos Santos P, Schoonderwoerd KM, Sontag S, Soulebeau A, Staedler Y, Tschan GF, Wing-Sze Leung A, Schonenberger J (2017) The ancestral flower of angiosperms and its early diversification. Nat Commun 8:16047. 10.1038/ncomms16047 28763051 10.1038/ncomms16047 PMC5543309

[CR61] Sherman BT, Hao M, Qiu J, Jiao X, Baseler MW, Lane HC, Imamichi T, Chang W (2022) DAVID: a web server for functional enrichment analysis and functional annotation of gene lists (2021 update). Nucleic Acids Res 50(W1):W216–W221. 10.1093/nar/gkac194 35325185 10.1093/nar/gkac194 PMC9252805

[CR62] Shirley BW, Kubasek WL, Storz G, Bruggemann E, Koornneef M, Ausubel FM, Goodman HM (1995) Analysis of Arabidopsis mutants deficient in flavonoid biosynthesis. Plant J Cell Mol Biol 8(5):659–671. 10.1046/j.1365-313x.1995.08050659.x 10.1046/j.1365-313x.1995.08050659.x 8528278

[CR63] Soltis PS, Soltis DE (2014) Flower diversity and angiosperm diversification. Methods Mol Biol 1110:85–102. 10.1007/978-1-4614-9408-9_4 24395253 10.1007/978-1-4614-9408-9_4

[CR64] Song C, Wang Y, Manzoor MA, Mao D, Wei P, Cao Y, Zhu F (2022) In-depth analysis of genomes and functional genomics of orchid using cutting-edge high-throughput sequencing. Front Plant Sci 13:1018029. 10.3389/fpls.2022.1018029 36212315 10.3389/fpls.2022.1018029 PMC9539832

[CR65] Takashi Nakatsuka K-iM, Abe Y, Kubota A, Kakizaki Y, Yamamura S, Masahiro Nishihara (2008) Flower color modification of gentian plants by RNAi-mediated gene silencing. Plant Biotechnol 25(1):61–68

[CR66] Teo ZWN, Zhou W, Shen L (2019) Dissecting the function of MADS-Box transcription factors in orchid reproductive development. Front Plant Sci 10:1474. 10.3389/fpls.2019.01474 31803211 10.3389/fpls.2019.01474 PMC6872546

[CR67] Theissen G, Saedler H (2001) Plant biology. Floral quartets. Nature 409(6819):469–471. 10.1038/35054172 11206529 10.1038/35054172

[CR68] Tsai WC, Dievart A, Hsu CC, Hsiao YY, Chiou SY, Huang H, Chen HH (2017) Post genomics era for orchid research. Bot Stud 58(1):61. 10.1186/s40529-017-0213-7 29234904 10.1186/s40529-017-0213-7 PMC5727007

[CR70] Valoroso MC, De Paolo S, Iazzetti G, Aceto S (2017) Transcriptome-wide identification and expression analysis of DIVARICATA- and RADIALIS-Like genes of the mediterranean orchid orchis Italica. Genome Biol Evol. 10.1093/gbe/evx101 10.1093/gbe/evx101 PMC5499889 28541415

[CR69] Valoroso MC, Censullo MC, Aceto S (2019a) The MADS-box genes expressed in the inflorescence of Orchis Italica (Orchidaceae). PLoS ONE 14(3):e0213185. 10.1371/journal.pone.0213185 30822337 10.1371/journal.pone.0213185 PMC6396907

[CR72] Valoroso MC, Sobral R, Saccone G, Salvemini M, Costa MMR, Aceto S (2019b) Evolutionary conservation of the orchid MYB transcription factors DIV, RAD, and DRIF. Front Plant Sci 10:1359. 10.3389/fpls.2019.01359 31736999 10.3389/fpls.2019.01359 PMC6838138

[CR71] Valoroso MC, Lucibelli F, Aceto S (2022) Orchid NAC transcription factors: a focused analysis of CUPULIFORMIS genes. Genes. 10.3390/genes13122293 10.3390/genes13122293 PMC9777940 36553560

[CR74] Wang N, Huo YX (2022) Using genome and transcriptome analysis to elucidate biosynthetic pathways. Curr Opin Biotechnol 75:102708. 10.1016/j.copbio.2022.102708 35278747 10.1016/j.copbio.2022.102708

[CR75] Wang Y, Shi Y, Li K, Yang D, Liu N, Zhang L, Zhao L, Zhang X, Liu Y, Gao L, Xia T, Wang P (2021) Roles of the 2-oxoglutarate-dependent dioxygenase superfamily in the flavonoid pathway: a review of the functional diversity of F3H, FNS I, FLS, and LDOX/ANS. Molecules. 10.3390/molecules26216745 10.3390/molecules26216745 PMC8588099 34771153

[CR76] Wang YS, Zheng W, Jiang N, Jin YX, Meng ZK, Sun MX, Zong YL, Xu T, Zhu J, Tan RX (2022) Alteration of the catalytic reaction trajectory of a vicinal oxygen chelate enzyme by directed evolution. Angew Chem 61(26):e202201321. 10.1002/anie.202201321 35415958 10.1002/anie.202201321

[CR73] Wang F, Deng Y, Pan Y, Wang S, Duan Y, Xue L, Wu X, Jiang Y, Chen J, Peng D (2025) Transcriptomics profiling and WGCNA provide new insights into anthocyanin metabolism in spring orchid (*Cymbidium goeringii*) flowers for future industrial applications. Ind Crops Prod. 10.1016/j.indcrop.2025.122240

[CR78] Wong DCJ, Amarasinghe R, Rodriguez-Delgado C, Eyles R, Pichersky E, Peakall R (2017) Tissue-specific floral transcriptome analysis of the sexually deceptive orchid chiloglottis trapeziformis provides insights into the biosynthesis and regulation of its unique UV-B dependent floral volatile, chiloglottone 1. Front Plant Sci 8:1260. 10.3389/fpls.2017.01260 28769963 10.3389/fpls.2017.01260 PMC5515871

[CR77] Wong DCJ, Amarasinghe R, Pichersky E, Peakall R (2018) Evidence for the involvement of fatty acid biosynthesis and degradation in the formation of insect sex pheromone-mimicking chiloglottones in sexually deceptive chiloglottis orchids. Front Plant Sci 9:839. 10.3389/fpls.2018.00839 29971087 10.3389/fpls.2018.00839 PMC6018206

[CR80] Yang F, Lu C, Wei Y, Wu J, Ren R, Gao J, Ahmad S, Jin J, Xv Y, Liang G, Zhu G (2021) Organ-specific gene expression reveals the role of the cymbidium ensifolium-mir396/growth-regulating factors module in flower development of the orchid plant Cymbidium ensifolium. Front Plant Sci 12:799778. 10.3389/fpls.2021.799778 35154190 10.3389/fpls.2021.799778 PMC8829051

[CR79] Yang D, Chen Y, He Y, Song J, Jiang Y, Yang M, Zheng X, Wang L, Hu H (2025) Transcriptome analysis reveals association of E-Class AmMADS-Box genes with petal malformation in *Antirrhinum majus* L. Int J Mol Sci. 10.3390/ijms26094450 10.3390/ijms26094450 PMC12072684 40362686

[CR83] Zhang L, Wang L, Yang Y, Cui J, Chang F, Wang Y, Ma H (2014) Analysis of Arabidopsis floral transcriptome: detection of new florally expressed genes and expansion of Brassicaceae-specific gene families. Front Plant Sci 5:802. 10.3389/fpls.2014.00802 25653662 10.3389/fpls.2014.00802 PMC4299442

[CR82] Zhang GQ, Xu Q, Bian C, Tsai WC, Yeh CM, Liu KW, Yoshida K, Zhang LS, Chang SB, Chen F, Shi Y, Su YY, Zhang YQ, Chen LJ, Yin Y, Lin M, Huang H, Deng H, Wang ZW, Zhu SL, Zhao X, Deng C, Niu SC, Huang J, Wang M, Liu GH, Yang HJ, Xiao XJ, Hsiao YY, Wu WL, Chen YY, Mitsuda N, Ohme-Takagi M, Luo YB, Van de Peer Y, Liu ZJ (2016) The Dendrobium catenatum Lindl. genome sequence provides insights into polysaccharide synthase, floral development and adaptive evolution. Sci Rep 6:19029. 10.1038/srep19029 26754549 10.1038/srep19029 PMC4709516

[CR81] Zhang GQ, Liu KW, Li Z, Lohaus R, Hsiao YY, Niu SC, Wang JY, Lin YC, Xu Q, Chen LJ, Yoshida K, Fujiwara S, Wang ZW, Zhang YQ, Mitsuda N, Wang M, Liu GH, Pecoraro L, Huang HX, Xiao XJ, Lin M, Wu XY, Wu WL, Chen YY, Chang SB, Sakamoto S, Ohme-Takagi M, Yagi M, Zeng SJ, Shen CY, Yeh CM, Luo YB, Tsai WC, Van de Peer Y, Liu ZJ (2017) The Apostasia genome and the evolution of orchids. Nature 549(7672):379–383. 10.1038/nature23897 28902843 10.1038/nature23897 PMC7416622

[CR84] Zhao X, Li Y, Zhang MM, He X, Ahmad S, Lan S, Liu ZJ (2023) Research advances on the gene regulation of floral development and color in orchids. Gene 888:147751. 10.1016/j.gene.2023.147751 37657689 10.1016/j.gene.2023.147751

[CR85] Zuntini AR, Carruthers T, Maurin O, Bailey PC, Leempoel K, Brewer GE, Epitawalage N, Francoso E, Gallego-Paramo B, McGinnie C, Negrao R, Roy SR, Simpson L, Toledo Romero E, Barber VMA, Botigue L et al (2024) Phylogenomics and the rise of the angiosperms. Nature 629(8013):843–850. 10.1038/s41586-024-07324-0 38658746 10.1038/s41586-024-07324-0 PMC11111409

