# Supplementary material for: Modular control of orchid beauty: co-expression networks orchestrate organ development and evolution in Phalaenopsis flower
Source: Plant Mol Biol. 2026 May 28;116(3):57. doi: 10.1007/s11103-026-01711-z (PMC13219088; doi:10.1007/s11103-026-01711-z)
Supplement: Supplementary file 1 — Supplementary List [file 11103_2026_1711_MOESM1_ESM.docx]

**Table S1** List of the genes from the WGCNA modules

**Table S2** Functional clusters of genes belonging to the WGCNA modules

**Table S3** List of gene-specific primer pairs used in the qPCR experiments

**Table S4** Conserved transcription factors binding sites found in the region of the putative promoters of genes belonging to the cluster 1 of the greenyellow module

**Table S5** Statistical significance of the difference in expression between the inner tepals and the lip components of wild-type *Phalaenopsis aphrodite* for the analyzed genes selected from the WGCNA modules

**Fig. SF1** Genes differentially expressed in the flower bud tissues of *Phalaenopsis aphrodite***.** (a) Heat map of the genes differentially expressed in *Phalaenopsis* floral tissues; (b) correlation matrix between the *Phalaenopsis* floral tissues; Biological Coefficient of Variation (BCV) between (c) column, ovary and perianth that comprises outer tepals, inner tepals, callus, lateral lobes (Lob_lat), central lobes (Lob cen); (d) outer tepals (Te_out) , inner tepals (Te_in) and lip that comprises callus, lateral lobes (Lob_lat), central lobe (Lob cen); (e) callus, lateral lobes (Lob_lat), central lobe (Lob cen)

**Fig. SF2** Principal component analysis (PCA) between (a) column, ovary and perianth that comprises outer tepals, inner tepals, callus, lateral lobes (Lob_lat), central lobe (Lob cen); (b) outer tepals (Te_out) , inner tepals (Te_in) and lip that comprises callus, lateral lobes (Lob_lat), central lobe (Lob cen); (c) callus, lateral lobes (Lob_lat), central lobe (Lob cen)

**Fig. SF3** Biological process enriched in WGCNA modules. These graphs represent the results of the Gene Ontology (GO) enrichment analysis, specifically focusing on the Biological Process (BP) category level, for the genes contained within co-expression modules. This analysis reveals which biological functions are statistically overrepresented in each module. On the x-axis we have the number of genes and on the y-axis the GO terms
