# Supplementary material for: Modular control of orchid beauty: co-expression networks orchestrate organ development and evolution in Phalaenopsis flower
Source: Plant Mol Biol. 2026 May 28;116(3):57. doi: 10.1007/s11103-026-01711-z (PMC13219088; doi:10.1007/s11103-026-01711-z)
Supplement: Supplementary file 6 — Supplementary Material SF2 [file 11103_2026_1711_MOESM6_ESM.docx]

**
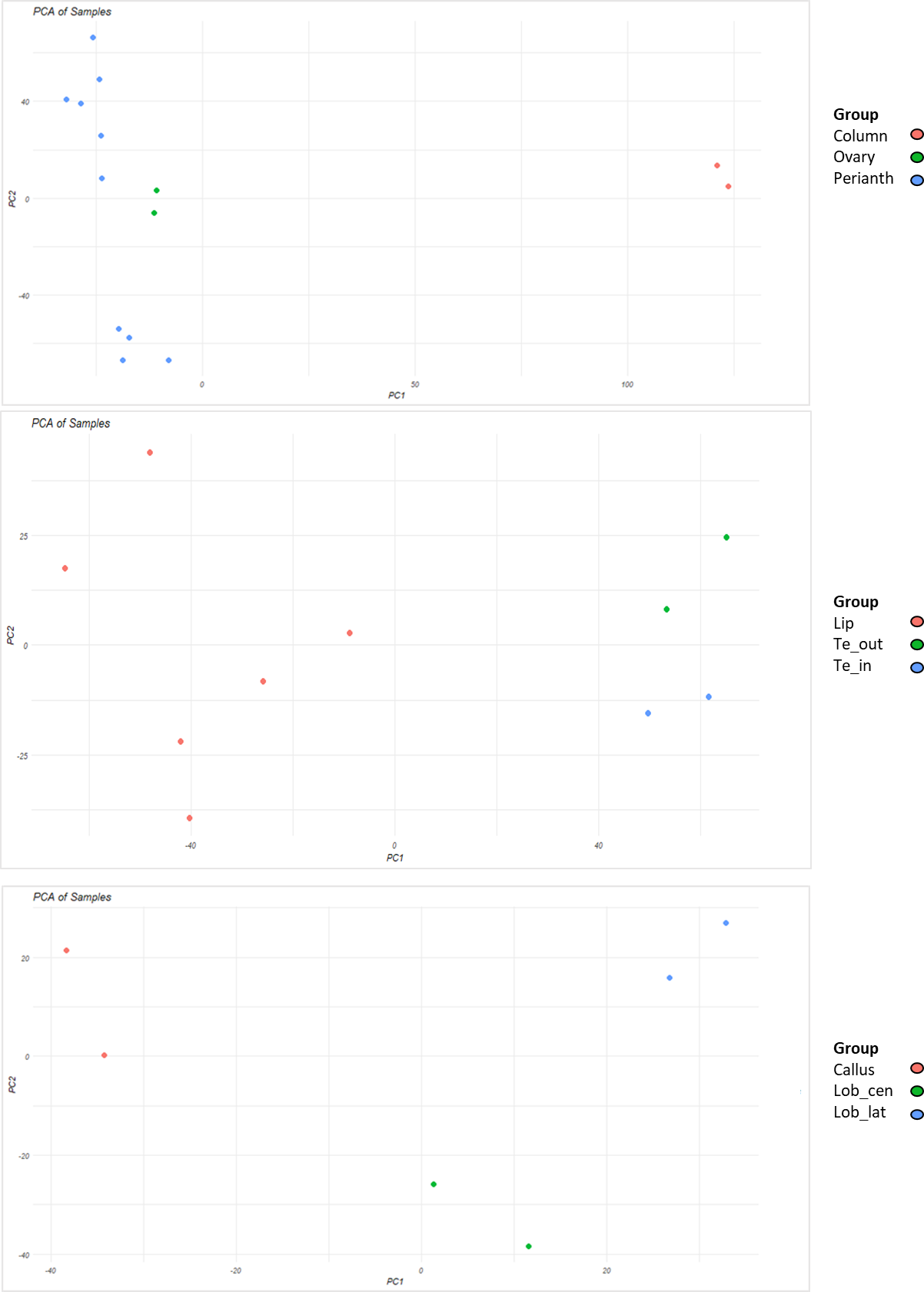
**

**Fig. SF2** Principal component analysis (PCA) between (a) column, ovary and perianth that comprises outer tepals, inner tepals, callus, lateral lobes (Lob_lat), central lobe (Lob cen); (b) outer tepals (Te_out) , inner tepals (Te_in) and lip that comprises callus, lateral lobes (Lob_lat), central lobe (Lob cen); (c) callus, lateral lobes (Lob_lat), central lobe (Lob cen)
