# Supplementary material for: Modular control of orchid beauty: co-expression networks orchestrate organ development and evolution in Phalaenopsis flower
Source: Plant Mol Biol. 2026 May 28;116(3):57. doi: 10.1007/s11103-026-01711-z (PMC13219088; doi:10.1007/s11103-026-01711-z)
Supplement: Supplementary file 7 — Supplementary Material SF3 [file 11103_2026_1711_MOESM7_ESM.docx]

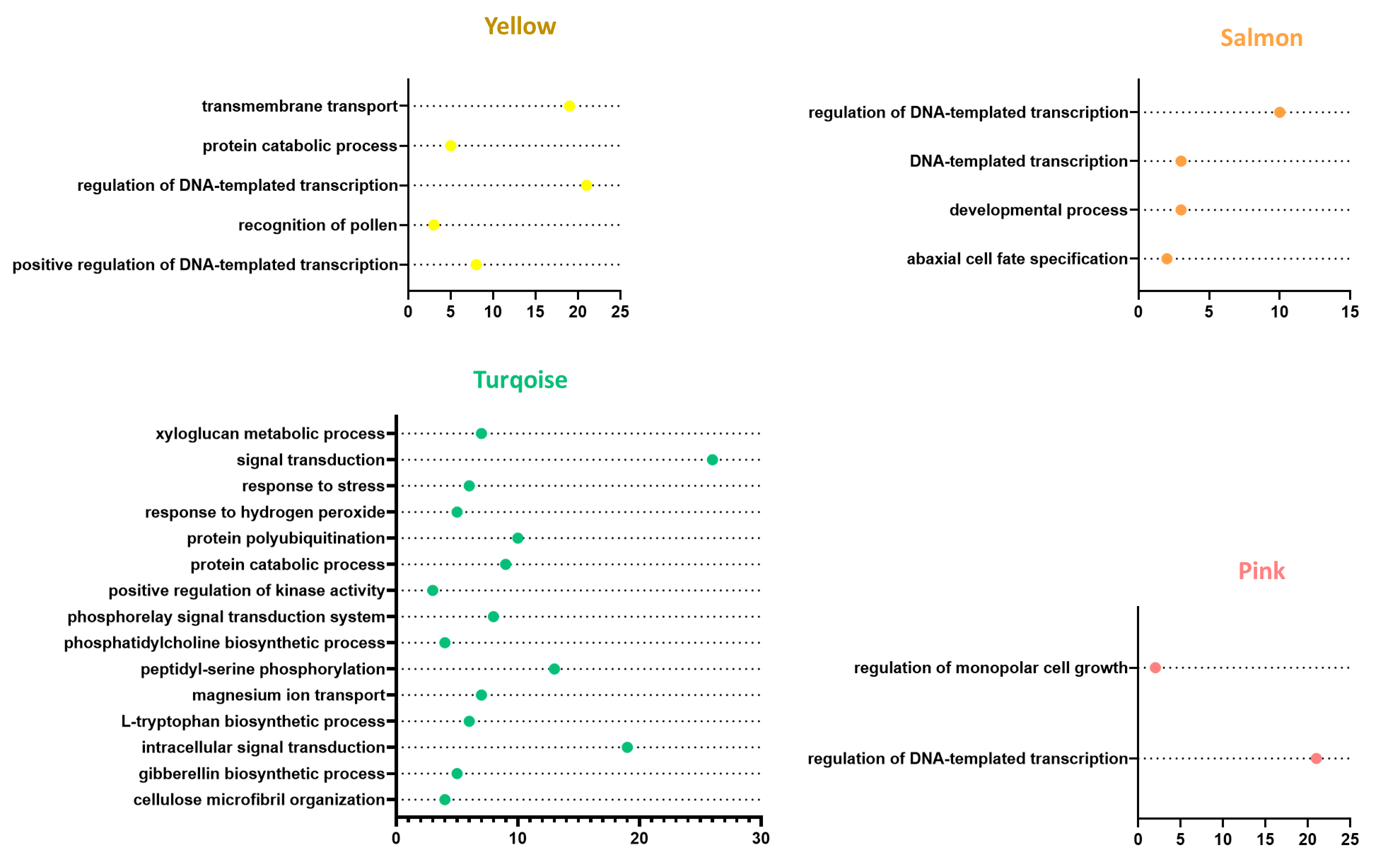


**Fig. SF3** Biological process enriched in WGCNA modules. These graphs represent the results of the Gene Ontology (GO) enrichment analysis, specifically focusing on the Biological Process (BP) category level, for the genes contained within co-expression modules. This analysis reveals which biological functions are statistically overrepresented in each module. On the x-axis we have the number of genes and on the y-axis the GO terms
