# Supplementary material for: Modular control of orchid beauty: co-expression networks orchestrate organ development and evolution in Phalaenopsis flower
Source: Plant Mol Biol. 2026 May 28;116(3):57. doi: 10.1007/s11103-026-01711-z (PMC13219088; doi:10.1007/s11103-026-01711-z)
Supplement: Supplementary file 8 — Supplementary Material SF1 [file 11103_2026_1711_MOESM8_ESM.docx]

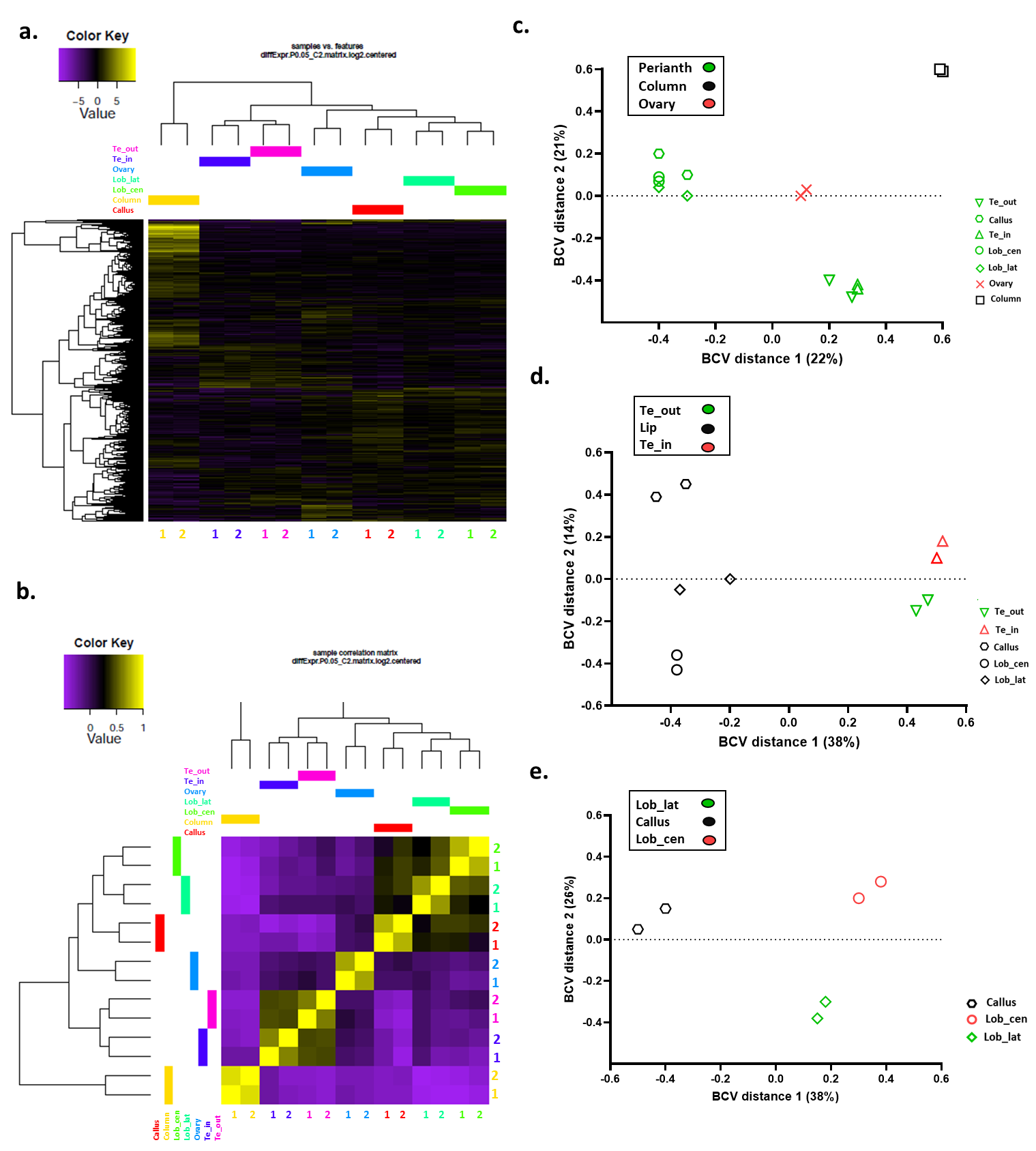


**Fig. SF1** Genes differentially expressed in the flower bud tissues of *Phalaenopsis aphrodite***.** (a) Heat map of the genes differentially expressed in *Phalaenopsis* floral tissues; (b) correlation matrix between the *Phalaenopsis* floral tissues; Biological Coefficient of Variation (BCV) between (c) column, ovary and perianth that comprises outer tepals, inner tepals, callus, lateral lobes (Lob_lat), central lobes (Lob cen); (d) outer tepals (Te_out) , inner tepals (Te_in) and lip that comprises callus, lateral lobes (Lob_lat), central lobe (Lob cen); (e) callus, lateral lobes (Lob_lat), central lobe (Lob cen)
